# Two-stage motion artefact reduction algorithm for electrocardiogram using weighted adaptive noise cancelling and recursive Hampel filter

**DOI:** 10.1371/journal.pone.0207176

**Published:** 2018-11-20

**Authors:** Fuad A. Ghaleb, Maznah Bte Kamat, Mazleena Salleh, Mohd Foad Rohani, Shukor Abd Razak

**Affiliations:** 1 Faculty of Engineering, School of Computing, Universiti Teknologi Malaysia, Johor, Malaysia; 2 Department of Engineering, Computer and Electronics Engineering, Sana’a Community College, Sana’a, Yemen; Indiana University, UNITED STATES

## Abstract

The presence of motion artefacts in ECG signals can cause misleading interpretation of cardiovascular status. Recently, reducing the motion artefact from ECG signal has gained the interest of many researchers. Due to the overlapping nature of the motion artefact with the ECG signal, it is difficult to reduce motion artefact without distorting the original ECG signal. However, the application of an adaptive noise canceler has shown that it is effective in reducing motion artefacts if the appropriate noise reference that is correlated with the noise in the ECG signal is available. Unfortunately, the noise reference is not always correlated with motion artefact. Consequently, filtering with such a noise reference may lead to contaminating the ECG signal. In this paper, a two-stage filtering motion artefact reduction algorithm is proposed. In the algorithm, two methods are proposed, each of which works in one stage. The weighted adaptive noise filtering method (WAF) is proposed for the first stage. The acceleration derivative is used as motion artefact reference and the Pearson correlation coefficient between acceleration and ECG signal is used as a weighting factor. In the second stage, a recursive Hampel filter-based estimation method (RHFBE) is proposed for estimating the ECG signal segments, based on the spatial correlation of the ECG segment component that is obtained from successive ECG signals. Real-World dataset is used to evaluate the effectiveness of the proposed methods compared to the conventional adaptive filter. The results show a promising enhancement in terms of reducing motion artefacts from the ECG signals recorded by a cost-effective single lead ECG sensor during several activities of different subjects.

## Introduction

An electrocardiogram (ECG) is a technique for diagnosis of cardiovascular diseases [1], and the ECG signal carries vital information about many cardiovascular disorders. It also can be used for diagnosing obstructive sleep apnoea, evaluating therapeutic drugs, and physiological monitoring. Early diagnosis of cardiovascular disease is necessary for the countermeasures and the remedial actions needed to save the patients. However, late diagnosis or misinterpretation of such information can lead to wrong medical decisions. Real-time monitoring or near to real-time monitoring is important for many patients who require long-term monitoring. A portable ECG device is beneficial for ambulatory monitoring, such as when monitoring the effect of drugs on the cardiac activities or post-surgical patients and during human normal activities [2]. The purpose of ambulatory monitoring is to capture the cardiac activity of the heart taking place while the user (subject) is performing all kinds of routine physical activities. However, the signals from most of the mobile (or wearable) ECG electrodes are generally corrupted by several types of noise, such as electromagnetic interference, muscle artefacts, baseline wander, and motion artefacts [3–7]. ECG noise reduction needs different strategies for different sources. Most of these noises can be effectively removed in real time by the application of digital filters. However, motion artefacts are difficult to remove without affecting the ECG signal itself.

Motion artefact is reported as the most difficult kind of noise to eliminate from ECG signals. The reason for this is that the spectrum of the motion artefact completely overlaps that of the ECG signal. In addition, its morphology resembles that of the P, QRS, and T waves [2]. Therefore, accurate rhythm assessment will be difficult to detect due to its similarity with the motion artefact. In addition, motion artefacts will cause a high false positive for some cardiovascular diseases, such as arterial fibrillation assessment in automatic healthcare systems [8]. Moreover, motion artefacts may also lead to misinterpretation of important information about a subject’s health status [5].

A motion artefact is a result of the change in the impedance between the skin and the electrode, which causes a transient change in the baseline signal [3]. The change in the baseline could be slow or quick depending on the severity of the electrode’s relative movement with the skin. For example, the skin under the electrode expands or shrink according to the weather condition which causes a reduction in the magnitude of the skin potential and thus the occurrence of the motion artefact. Besides, the electrode may slide due to the sweating causing the electrode loss the contact with the skin which led to the appearance of the motion artefact. Moreover, the subject’s motion can morphologically damage the ECG signal due to the electrode displacement (loose contact and slide) and the physical condition of the skin, such as dryness and wetness. For example, the electrode may be displaced from the skin due to the subject of sweating or skin stretching. If the skin stretches, the potential between the skin and electrode changes, causing transient ECG change [5, 9, 10]. In many cases, the spectrum of the motion artefact overlaps with the ECG signal when the subject is walking or moving. Thus, it is very difficult to remove the motion artefacts from an ECG signal [11].

Reducing motion artefacts has been the subject of several studies, in the context of Digital Signal Processing (DSP) such as Discrete Wavelet Transform (DWT) [12–14], Adaptive Digital Filtering (ADF) [2, 10, 15], and the statistical procedures such as Independent or Principal Component Analysis (ICA or PCA) [16–18]. However, these techniques have shortcomings that make them unsuitable for the mobile ECG environment and its expected types of noise. Independent component analysis (ICA) has shown some advantages for reducing motion artefact noises. However, ICA needs many leads in which the sources of noise are independent from the ECG source but correlated with each other. These conditions may be rarely met in reality during portable ECG recording because motion artefact noise seems to be dependent on the electrode coverage area. The impedance has spatial distribution on the skin, thus ICA can solve the issue only partially. Similarly, the adaptive noise cancelling approach has received attention from researchers, due to its effectiveness in removing motion artefacts if a noise reference is available. However, the existing studies assume that the noise in the ECG signal correlates directly with the movement [19–21], which is not really accurate in resembling motion artefact noise. Electrode motion does not necessarily have a high correlation with the body movement, because the body could move while the electrode is still attached to the body, and thus the change in the skin impedance does not necessarily result from electrode movement.

In this paper, a two-stage filtering algorithm is proposed. In the first stage of the proposed algorithm, a weighted adaptive noise cancelling method (WAF) is proposed. The acceleration of the electrode is used as noise reference, while the correlation between the acceleration and the recorded ECG is used as an updating weight factor. The acceleration signals were recorded using triaxial accelerometer integrated with the ECG electrode. The noise reference intensity is modified accordingly to the correlation with the acceleration. Thus, the impact of the adaptive noise cancelling algorithm on the ECG signal is decreased during low correlation conditions. In the second stage, a statistical -based filter is used for estimating the potential ECG signal. The recursive Hampel filter-based estimation method (RHFBE) is proposed for estimating the ECG structure. The Hampel filter [22] is used due to its robustness against outliers. The ECG signal is segmented i.e. each cardiac cycle, namely, P-QRS-T is separated in one segment. Thus a set of successive segments is used for the estimation, based on the Hampel filter, which is considered a conditional median-based filter. The results show a potential enhancement in the reduction of the motion noise from ECG signals recorded during a subject’s normal activities using Holter recording. The performance of the proposed algorithm is compared to the adaptive noise cancelling, which is widely suggested for motion artefact removal in many studies.

The main contributions in this research are summarized as follow.

1. A two-stage motion artefact filtering algorithm is proposed to address the issue of adaptive noise filter due to the occasional and variable correlation between acceleration and ECG signal. A new filtering concept has been introduced which is the one-lead based spatial correlation between the successive ECG cycles (P-QRS-T segments). While the successive ECG cycles have similar characteristics, the motion artefact is random in the successive cardiovascular cycle. Therefore, the spatial correlation between the successive P-QRS-T segments has been exploited to remove the motion artefact.
2. A Weighted Adaptive noise Filtering method (WAF) is proposed to address the impact of the occasional and low correlation between the ECG and the acceleration signal on the filtered ECG signal. The effectiveness of the proposed method over the conventional adaptive filter method has been shown.
3. A R-peak Annotation and Signal Segmentation method (RAS) is proposed that use effective QRS detection algorithms to detect and annotate the R-peak. It worth mentioning that the use of the adaptive filter prior to the proposed segmentation method improves the QRS detection accuracy [23]. Moreover, the detection of the R-peak is simpler than detecting and removing the motion artefact. Thus, QRS detection algorithm is used as a pre-step before removing the motion artefact in some studies such as in [24]. Therefore, a QRS detection algorithm is applied on the output of the proposed weighted adapted filter and used as preprocessing step before applying the spatial filter in the next phase of the proposed algorithm.
4. A novel spatial filter is proposed namely the recursive Hampel filter-based estimation method (RHFBE) which assumes that the motion artefact in the new P-QRS-T segment is far from the median of the successive P-QRS-T segments. Hampel filter is a robust statistical filter for removing outliers thus it is considered effective and efficient for real-time applications [22]. The difference between Hampel filter and the conventional median filter is that Hampel filter can be considered as a conditional median-based filter which replaces only the outlier sample which exceeds specific threshold called absolute median deviation, while median filter replaces all the samples by the median. Thus, the conventional median filter cause filter out some disease patterns. Consequently, the analyst fails to detect certain diseases cases. Meanwhile, Hampel filter can preserve the information as it only replaces the outlier that is far enough from the median. The effectiveness of the Hampel filter based algorithms over the conventional adaptive filter based algorithm has been shown.

The rest of the paper is organized as follows. In section two, the related work is discussed. In Section three, the preliminary information that needed for the design of the proposed algorithm is briefly described. The proposed algorithm is explained in Section four. The performance evaluation and results are discussed in Section five and six, respectively. The conclusions are drawn in Section seven.

## Related work

Traditional digital signal processing filters such as band-pass filter, and Discrete Wavelet Transform (DWT) [12–14] are useless for removing motion artefacts from a contaminated ECG signal, due to the overlapping of the motion artefact with the desired ECG signal [25, 26]. According to [27] the bandwidth of a motion artefact is less than 5 Hz and that it can be filtered through oversampling, such as 2 KHz, for better time resolution. However, the use of a conventional digital filter such as a stop-band or high pass filter can remove a portion of the ECG signal such as P or T waves. Many alternative techniques have been proposed for cancelling motion artefacts, such as Independent Component Analysis (ICA) [28–32], Empirical Mode Decomposition (EMD) [8, 33–36] and Adaptive Noise Cancelling [2, 21, 37–40]. ICA assumes that the ECG and motion artefact sources are independent. Accordingly, blind separation is applied for separating the noise from the ECG. This relation is true between ECG and motion artefacts; however, ICA also assumes that all ECG channel signals are contaminated from the same source of noise i.e. there is only one source of motion artefact that affects all ECG channels. This assumption is not accurate, due to the spatial distribution of the skin impedance under the electrode sensors. Moreover, ICA is rarely suggested for mobile ECG recording due to the resource limitations of mobile ECG. Empirical Mode Decomposition (EMD) is another effective way for reducing motion artefact noise. However, it is difficult to identify which empirical member needs to be removed from the signal decomposition. Adaptive Noise Cancelling (ANC) has received increasing attention from researchers in the last few years [3, 4, 39–42] due to its suitability for real-time environments. Moreover, an adaptive filter is effective if the reference signal for the noise is available with high correlation with the noise in the ECG signal.

In the field of adaptive noise cancelling, [2] proposed an adaptive Recursive filter for motion artefact noise cancellation (ANC) in which the reference signal is an impulse train coincident with the QRS complexes. However, they reported that the proposed filter can reduce the motion artefact only if the motion artefact is stationary and coincident with the QRS complex. Thus, the motion artefact which affected the QRS complex part was removed, while the motion artefact that impacted the other parts of the ECG signal has not been treated. Moreover, they also reported that using the impulse train as reference distorts the P wave and T wave part in the ECG.

Tong et al., [15] proposed an adaptive filter that utilized the electrode motion as the reference of the motion artefact. Two types of sensors were used, namely an anisotropic magneto resistive sensor (AMR) and a three-axis accelerometer sensor (ACC), to measure the electrode motion to be used as the reference of the motion artefact. The authors reported that the ACC sensor produced better results than AMR, for two possible reasons: (i) ACC gives measurements for three dimensions, whereas AMR provides only two dimensions, and (ii) the ACC measurement modelled the electrode motion more accurately than the AMR measurement model. Although the use of electrode motion as a reference for noise is considered effective for clinical recording, it is very difficult to remove the motion artefact in mobile ECG due to vigorous movement by the subject.

Yoon et al., in [19] used the difference between two types of ECG electrodes, namely Ag/AgCl and e-textile, as a desired motion artefact signal, while a 3-axis accelerometer sensor was used as the reference for the motion artefact. The adaptive filter was used to accurately estimate the motion artefact noise. The output of the filter was subtracted from the e-textile electrode records. However, it is not clear why two types of ECG were used for representing the desired signal of the motion artefact. In addition, there are no details provided about the effectiveness of the proposed filtering method.

Liu in [3] proposed an adaptive filter where a triaxle accelerometer was used as the motion artefact noise signal and reported that the performance of the filters was directly related to the correlation of the reference signal with the noise in the ECG data. However, the author also reported that some elements of the ECG signal, such as P-waves and T-waves, were totally contaminated. In addition, it can be noted that the most part of the noise still presented in the filtered signal.

Zhang et al., in [42] investigated the use of the adaptive filter for reducing the motion artefact in wearable ECG electrodes during exercise. The triaxle accelerometer (ACC) was used as input to three gradient adaptive Laguerre lattice (GALL) filters to estimate the motion artefact. The outputs of the GALL filters were combined using a time-varying unforced Kalman filter. The authors concluded that the accelerometer as a reference signal cannot effectively reduce motion artefacts, due to the weak linear correlation between ACC and ECG noise.

Lee et al, in [8] used empirical mode decomposition for detecting motion artefacts in ECG data collected by a Holter recorder. The detection algorithm used the statistics of the square of the first-order intrinsic mode function to detect the corrupted portion of the ECG. The aim of the algorithm was to decrease the false positive of an automated atrial fibrillation detection system. The noise-contaminated ECG segments were ignored.

Kirst et al, in [25] proposed an algorithm for removing motion artefacts using discrete wavelet transform (DWT) for long-term monitoring. Considering subject comfort and skin tolerance, textile-based dry electrodes were used for data recording. The signal of the skin impedance sensor was compared to the signal of the acceleration sensor. The results of the comparison confirmed that ACC has low correlation with the disturbed ECG sequence, while electrode-skin impedance has a higher correlation. As the change in the impedance between the electrode and the skin is the origin of motion artefacts, the authors used electrode-skin impedance as a second channel for reducing motion artefacts. One important point that can be noted in their experiment is that ACC could also be correlated with the ECG itself, and not the noise in the ECG. Meanwhile, the skin-impedance has low correlation with the desired ECG. The proposed algorithm utilized the signal of a skin-impedance sensor to identify which portion of the signal was contaminated by the motion artefact. Four-level wavelets were applied on both the ECG and impedance signals. If one of the four-level signals of the impedance signal exceeded a predefined threshold, the corresponding sample on the ECG signal was set to zero (zeroing). The filtered signal was obtained by reconstructing the modified signals. The reported algorithm was validated using a QRS detection algorithm specificity. The authors reported that the artefact region may be destroyed due to wrong threshold selection.

It is common practice to measure the electrode-skin impedance prior to ECG recording [43]. However, if the current electrode-skin impedance mismatches the prior measurements, then the recorded signal will be corrupted by a motion artefact. Degen et al., in [43] proposed a method to monitor the skin stretch through optical stretch sensors. The motion artefact was removed through an adaptive noise canceler. However, the effectiveness of the proposed method has not yet been tested on real motion artefacts; only an artificial motion artefact was used for the evaluation.

Hughes and John in [9] investigated the impact of the skin-stretching on the impedance of different skin layers. It was revealed that the skin-impedance decreased from the outside to the inside. Moreover, the outside electrode-skin impedance was found to be dependent on the subject’s skin while this dependency decreases in the inner layers of the skin. The inner skin layers have more connectivity and less response to the skin stretching. However, in mobile ECG it is not feasible to strip away the skin in order to prevent the motion artefact resulting from skin stretching. Besides, the use of electrode-tissue impedance sensor is not comfortable by the users due to the needs of implanting the electrode under the subject’s skin.

Existing motion artefact solutions either cannot totally remove the motion artefact or they lead to significantly changing the ECG morphology. The application of adaptive noise filtering is the most reported solution for motion artefact reduction. However, an adaptive filter needs a reference signal that is highly correlated with the noise in the ECG signal and not with the ECG information. If the correlation is weak, the resultant filtering signal can be wrong. Both ECG and motion artefacts are difficult to obtain in reality and they have also time-variant characteristics; therefore they are not easy to estimate in practice. However, the advantage of using adaptive filters is that the reference signal is not necessary. The motion artefact filtered signal is only suitable for detecting the QRS complex or arrhythmia. ANC cannot totally remove the noise, as it is difficult to find a highly correlated noise reference. In contrast, the presence of uncorrelated noise in the reference signal degrades the performance of the ANC.

In this paper, a two-stage filtering algorithm is proposed for reducing motion artefacts from ECG signals recorded during normal subject activities. In each stage, a different filtering method is proposed. The first stage is to use the concept of an adaptive noise filter with acceleration as a reference and propose weighted adaptive noise filtering. The difference between this method and the existing adaptive noise filters is that the cross-correlation coefficient between the ECG and acceleration is used for modifying the adaptive filter weights. The noise is multiplied by a factor which is the cross-correlation coefficient between the raw ECG signal and the estimated noise signal through Least Mean Square (LMS) algorithm. Thus, the impact of the false reference signal is reduced, which lead to better resamples of the motion artefact. In the second filtering stage, a recursive Hampel filter-based estimation method (RHFBE) is proposed to further reduce the remaining motion artefact from the ECG signal which has low correlation with the acceleration signal. The Hampel filter is proposed for three main reasons. Firstly, the morphology of the ECG signals changes slowly with time, such that the successive ECG cycles have similar characteristics. Secondly, the motion artefact has a random impact on the ECG signal elements, i.e. the motion artefact is not repetitive in each cardiovascular cycle; therefore, the contaminated portion can be shown as an outlier with respect to the successive segments. Thirdly, the Hampel filter is a statistical filter outlier that is robust and thus it is considered efficient and effective for real-time applications. The Hampel filter is found to be fit for such a situation. Both the adaptive noise canceler and the Hampel filter are efficient and can work in real time.

## Preliminary

In this section, the preliminary information is presented in order to drive the proposed motion artefact reducing algorithm.

### Adaptive filter (AF)

**Fig 1:**
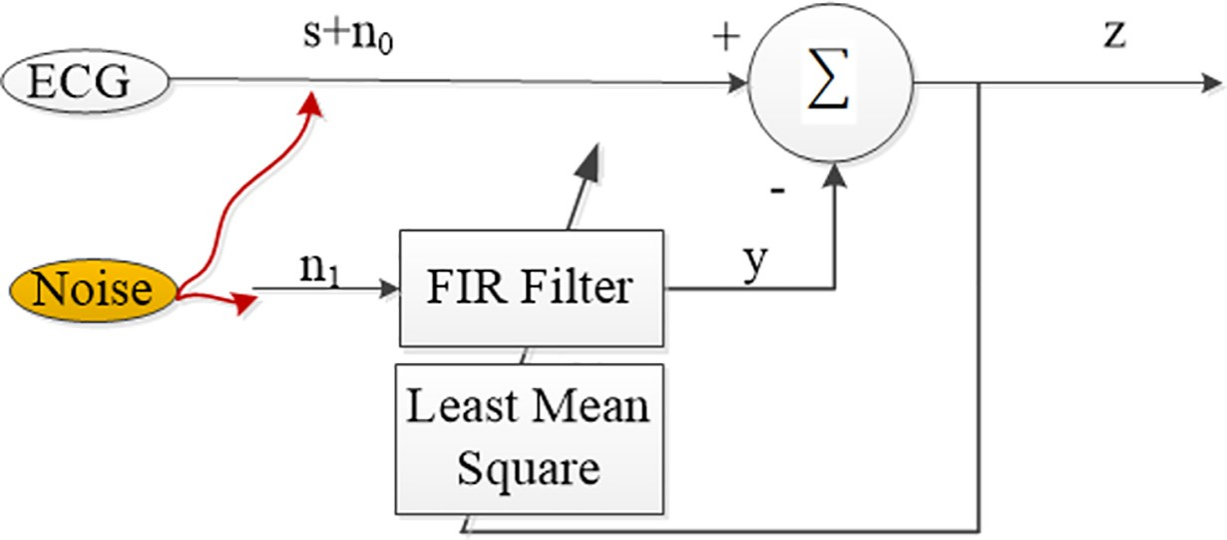
Adaptive noise filter concept.

The adaptive filter (AF) or the adaptive noise canceller (ANC) [37] is a filter for removing the noise from a signal that is contaminated with additive noise. It can take a reference input noise that is correlated with noise in the original signal in order to subtract the noise from the contaminated signal. Fig 1 illustrates the concept of ANC.

As shown in Fig 1, an adaptive noise filter requires two inputs: primary and reference. Let the primary input of the filter be the ECG signal (*s*_*ecg*+*ma*_ = *s*_*ecg*_+*n*_*ma*_) that is contaminated by the motion artefact while the reference is the noise signal (*n*_*r*_). The output of the adaptive filter is the estimated noise $\left(n_{ma}^{\prime }\right)$ and the residual of the difference between the ECG signal is $\varepsilon =s_{ecg+ma}-n_{ma}^{\prime }=s_{ecg}+n_{ma}-n_{ma}^{\prime }$, then:

$$\varepsilon^{2}={\left(s_{ecg}\right)}^{2}+2*s_{ecg}(n_{ma}-n_{ma}^{\prime })+{\left(n_{ma}-n_{ma}^{\prime }\right)}^{2}$$ (1)

By taking the mean of the square error of the equation above,

$$E[\varepsilon^{2}]={E[(s_{ecg})}^{2}]+E[2*s_{ecg}(n_{ma}-n_{ma}^{\prime })]+E[{\left(n_{ma}-n_{ma}^{\prime }\right)}^{2}]$$ (2)

Because the signal *s*_*ecg*_ and noise $\left(n_{ma}-n_{ma}^{\prime }\right)$ are uncorrelated, therefore,

$$E[\varepsilon^{2}]={E[(s_{ecg})}^{2}]+E[{\left(n_{ma}-n_{ma}^{\prime }\right)}^{2}]$$ (3)

To minimize the mean square error, the least mean square (LMS) algorithm can be used. The LMS tries to adaptively adjust the filter coefficient so that *n*_*ma*_ can be resampled by $n_{ma}^{\prime },$ and thus the square error difference ${\left(n_{ma}-n_{ma}^{\prime }\right)}^{2}$ is minimized. Let *W*_*k*_ = [*w*_1(*k*)_,*w*_2(*k*)_,*w*_3(*k*)_,…,*w*_*n*(*k*)_], where W_k_ is a vector which contains the filter’s coefficients at the time epoch *k*. The LMS algorithm can be written as follows:

$$W_{k+1}=W_{k}+2\mu \varepsilon_{k}x_{r(k)}$$ (4)

where *x*_*r*(*k*)_ is the reference noise and *X*_*r*(*k*)_ = [*x*_*r*1(*k*)_,*x*_*r*2(*k*)_,*x*_*r*3(*k*)_,…,*x*_*rn*(*k*)_], *ε*_*k*_ is the filtered clean signal and $\varepsilon_{k}=s_{ecg+ma}-n_{ma}^{\prime }$ and *μ* is the learning parameter, which can be calculated according to the following equation,

$$\mu_{k}=\frac{\beta }{\|x_{r(k)}\|+c}$$ (5)

where *β* is normalized step size (0<*β*<2), and *c* is a small positive constant that prevents *μ*_*k*_ from taking large value when *x*_*r*(*k*)_ is near zero. The estimated noise, $n_{ma(k)}^{\prime }$ is calculated with the following expression

$$n_{ma(k)}^{\prime }=W_{\left(k\right)}X_{r(k)}$$ (6)

where *W*_(*k*)_ is a vector which contains the filter’s coefficient, and *X*_*r*(*k*)_ is the reference noise.

### Hampel filter (HF)

The Hampel identifier is regarded as one of the most robust and efficient outlier identifiers [22]. The Hampel filter is based on a moving window for detecting outliers in a time series. Usually, HF is used as a robust alternative to the outlier sensitive z-score (three-sigma rule). Hampel replaces the mean and the standard deviation by the median and median absolute deviation (MAD) which are outlier-resistant parameters. The difference between HF and other median filters is that HF uses a scaling factor that takes the distribution of the data into account, while the other median filters use a constant threshold.

Given a sequence *X*_*k*_ = [*x*_1_,*x*_2_,…*x*_*l*_] with sliding window *l* at time epoch *k*, the median and MAD can be calculated as follows

$$\emptyset_{k}=median(X_{k})$$ (7)

$$\varphi_{k}=1.4826*median(x_{k}-\emptyset_{k})$$ (8)

After replacing the mean and the standard deviation by the median and MAD, the modified rule is written as follows:

$$|x_{k+1}-\emptyset_{k}|\geq m_{\sigma }\times \varphi_{k}$$ (9)

The HF identifier declares that *x*_*k*+1_ is an outlier and replaces it with the median ∅_*k*_ if it is located away from the *m*_*σ*_^*th*^ times the standard deviation.

### Detection of the QRS-complex

The QRS complexes in an electrocardiogram represent the depolarization phenomenon of the ventricles and yield useful information about their behaviour. Detecting the QRS complex is a very common procedure for analyzing ECG signals in healthcare systems. It is the first step in the automatic ECG processing algorithms. Real-time QRS complex detection is essential for monitoring of patients in critical heart condition. Therefore, many researchers in the QRS-Complex detection have focused on increasing the performance in terms of detection accuracy and computational complexity of the detection. The scope of this study is limited at utilizing one of the existing real-time QRS detection algorithms to label the R-peak locations in the ECG signal in order to accomplish accurate segmentation of the QRS complexes.

QRS complex detection is considered simple task and can be done efficiently for clinical ECG recording. Many effective techniques have been proposed for QRS detection algorithms, including using neural networks [4], the hidden Markov model (HMM), and wavelet transform (WT) [44]. Unfortunately, these techniques are inefficient for low-cost portable ECG and long-term monitoring as their use will consume more energy. However, detecting the QRS complex in mobile ECG is more challenging due to the number of disturbance sources, such as radio-based noises, subjects’ movement, and weather conditions, as well as the limited number of leads used for the recording. Hence, QRS detection in mobile ECG has been widely studied, and many complex and effective methods exist in the literature. The majority of the reported techniques are highly accurate, even under a harsh recording environment. However, most of these techniques work offline and also consume a considerable amount of resources, such as memory, computational time, and power. Fortunately, due to the dominant feature of the R-peak in the QRS complex, it can be distinctly recognized from its sharp edges and high amplitude. The high slope of the QRS complex motivates the employment of the temporal derivative of the ECG signal for QRS detection. Many methods have been proposed for real-time requirements, such as those proposed by authors in [33, 34], which showed their effectiveness and efficiency in detecting the QRS complex under different recording conditions in real time. Friesen et al, studied the noise sensitivity of nine different QRS detection algorithms [45]. In their study, the QRS detection algorithms were applied on ECG signals corrupted with many synthesized noise types. The study showed that algorithms based on amplitude and slope had the highest performance in terms of QRS detection accuracy. Therefore, QRS detection algorithms could be used as a preprocessing step for further improving the quality of the ECG signals [24]. Accordingly, the Pan and Tompkins QRS detection algorithm which is presented in [46] is selected for the segmentation phase in this study prior to spatial filtering using Hampel filter. Pan and Tompkins algorithm which operates according to the slope, amplitude and width, is the most reported accurate QRS detection method in the literature [35]. The results showed the sensitivity (SE) of this algorithm was of 99.75% with a positive predictive value (PPV) of 99.54%. However, any other efficient and effective QRS complex, such that of Pandit et al [47] and Kin and Chin [48] can also be used.

## The proposed algorithm

**Fig 2:**
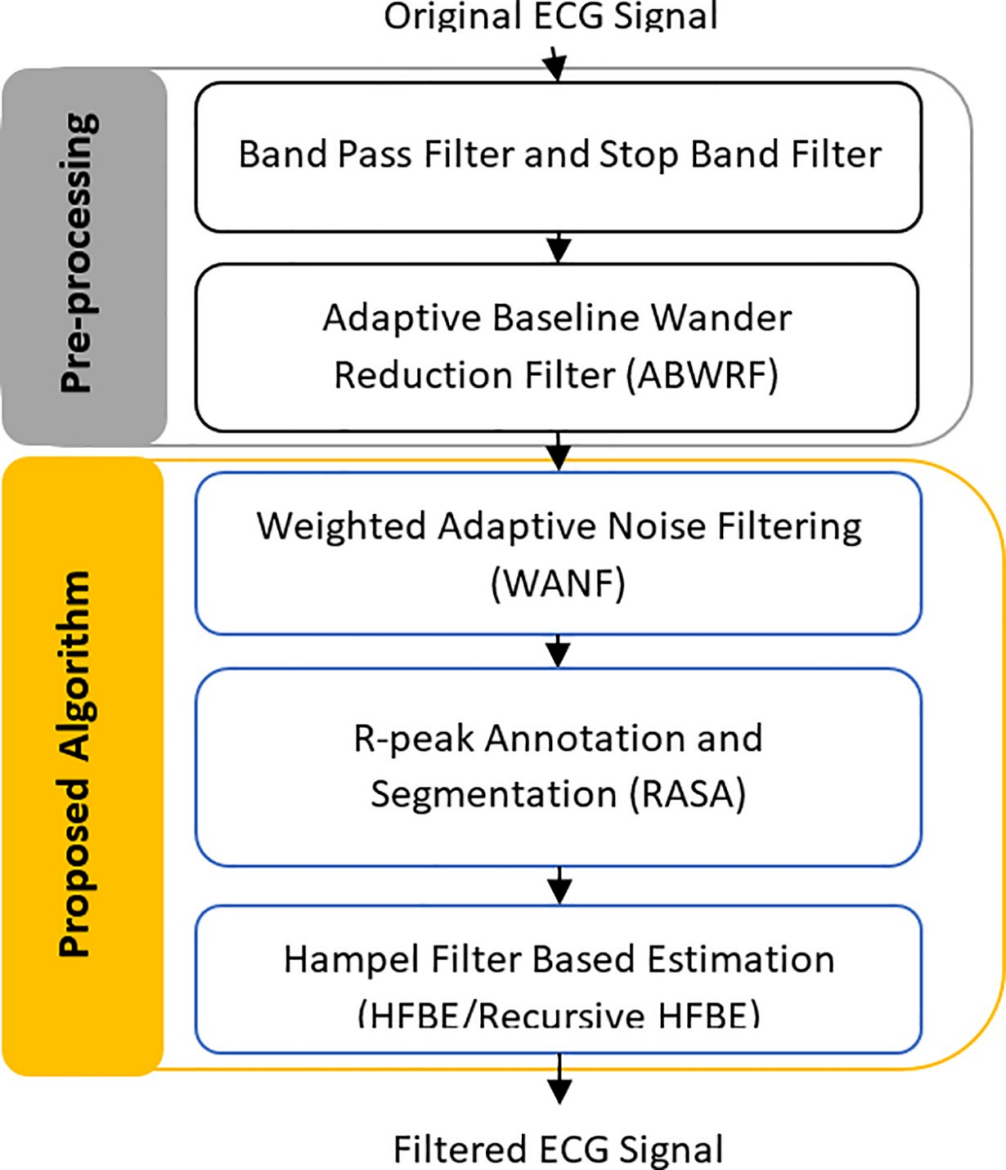
Block diagram of the proposed motion artefact reduction algorithm.

The aim of this section is to explain the proposed algorithm. The main goal of the algorithm is to reduce the motion artefact of an ECG signal acquired through portable or mobile ECG while a subject performs normal activities. The proposed algorithm consists of five modules, as shown in Fig 2. They are Bandpass Filter with an ECG normalization module, the Adaptive Baseline Wander Reduction Filter module (ABWRF), the Weighted Adaptive Noise Filter (WANF) module, the R-Peak Annotation and Segmentation Algorithm (RPASA) module, and the Hampel Filter-Based Estimation (RHFBE) module which contain both HFBE and the Recursive Hampel Filter-Based Estimation.

### Pre-processing module

The pre-processing phase consists of two parts, the Butterworth band-pass filter for removing high frequencies followed by notch filter for eliminating the 50/60Hz power line interference, and the adaptive noise canceller for removing baseline wandering. Industrial environment can be a source of radiation generated by high voltage power lines, transformers, welders, electric motors, induction furnaces, degaussing coils, etc. Thus, in the first part, the ECG signals are digitally filtered with a zero-phase Butterworth IIR bandpass filter (0.05–130 Hz). The notch filter is used as stopband to suppress the 50/60Hz power line interference.

The second part uses the adaptive baseline wandering reduction filter (ABWRF) proposed by [2]. The concept of reducing the baseline wander noise from the ECG signal involves removing the DC component from the ECG signal. Therefore, adaptive noise cancelling with one weight is considered effective for removing such noise from the signal. The reference input of the ABWRF is set to a constant with a value of one.

### Weighted adaptive filtering module (WAF)

The goal of the adaptive filter is to adapt the filter coefficient in order to estimate the motion artefact in the ECG signal. This filter is designed based on a conventional noise canceller (ANC) namely the adaptive filter (AF). The AF is modified to include the correlation between the noise reference and the ECG signal as a weight for adjusting the amount of filtering according to the correlation. Thus, the impact of the uncorrelated noise is reduced and a better ECG signal is obtained.

To prevent formation of incorrect $n_{ma(k)}^{\prime }$, the cross-correlation vector between the acceleration signal (*n*_*r*_) and the ECG signal (*s*_*ecg*_*ma*_) *ρ*_(*k*)_ = [*ρ*_1(*k*)_,*ρ*_2(*k*)_,*ρ*_3(*k*)_,…,*ρ*_*n*(*k*)_] is multiplied by the weights to reduce the impact of uncorrelated noise from disturbing the original signal, as follows:

$$\rho_{k}=\frac{\sum_{i=1}^{m}\left(n_{r(i)}-\mu (n_{r}))(s_{ecg\_ma(i)}-\mu (s_{ecg\_ma})\right)}{\sqrt{\sum_{i=1}^{m-1}{\left(n_{r(i)}-\mu (n_{r})\right)}^{2}\sum_{k=1}^{m}{\left(s_{ecg\_ma(i)}-\mu (s_{ecg\_ma})\right)}^{2}}}$$ (10)

$$n_{ma(k)}^{\prime }=W_{\left(k\right)}\rho_{\left(k\right)}X_{r(k)}$$

A triaxial accelerometer is attached to the ECG electrode and used as noise reference, namely the component of the triaxial record vector is compounded to on value so-called Signal Vector Magnitude (SVM). Similar to many previous research such as in [36, 37], the absolute value of the acceleration vector (SVM) is used as reference because its contain the change in the acceleration in the three directions and it can represent the acceleration signal at any direction. SVM is calculated as follows.

$${n_{r}=ACC}_{SVM}=\sqrt{{acc}_{x}^{2}+{acc}_{y}^{2}+{acc}_{y}^{2}}$$ (11)

where *n*_*r*_ is the noise reference and *ACC*_*SVM*_ is the SVM value of the recorded accelerations in the three axes, *x*, *y* and *z*, respectively.

### R-peak Annotation and Signal Segmentation Algorithm Module (RASA)

The output of the WAF algorithm is buffered. For each time epoch *k*, the QRS detection algorithm is then used to annotate the buffered signal. An indexing vector is created to mark the location of the QRS complex, namely the R spike, by setting the location to one. A window started at the R peak signal is then used for signal segmentation. The R-peak is selected in the beginning of the window inorder to address the issue of the irregulare heartbeat signal (Arrhythmia). A complete segment containing the P-QRS-T signal with fixed length is separated and stored in the matrix which contains L time-sequences as follows:

$$Buffer_{k\left(l,w\right)}=[\begin{array}{cccccc} S_{\left(k-l/2\right)} & S_{\left(k1-l/2+1\right)} & \cdots & S_{\left(k\right)} & \cdots & S_{\left(k+l/2\right)}\\ S_{\left(k1-l/2\right)} & S_{\left(k2-l/2+1\right)} & \cdots & S_{\left(k1-l\right)} & \cdots & S_{\left(k1+l/2\right)}\\ S_{\left(k2-l/2\right)} & S_{\left(k3-l/2+1\right)} & \cdots & S_{\left(k2-l\right)} & \cdots & S_{\left(k2+l/2\right)}\\ \vdots & \vdots & \vdots & \vdots & \vdots & \vdots \\ . & . & . & . & . & .\\ S_{\left(kw-l/2\right)} & S_{\left(kw-l/2+1\right)} & \cdots & S_{\left(kw-l\right)} & \cdots & S_{\left(kw+l/2\right)} \end{array}]$$

where *k* is the time epoch of complete P-QRS-T signal, which is the moment when the R peak occurred, *w* is the number of signals that were taken into account from successive ECG signals, and *l* is the length of the complete P-QRS-T ECG signal (it is also refered to buffer width in this paper). *w* is set according to the amount of time that the signal is stationary. With the assumption of an uncorrelated noise signal, *w* may be selected according to the interquartile range *IQR* length of each column in the *Buffer*_*k*(*l*,*w*)_. IQR is a measure of variability which is the difference between the first and the third quartile of each column in the *Buffer*_*k*(*l*,*w*)_. If all *IQRs* are small, this is an indication of a precise signal, so a small buffer size (*w*) is enough to estimate the correct ECG signal. Meanwhile, if the *IQRs* are large, then this is an indication of imprecise ECG signals; thus a larger buffer size, *w*, should be used. If a corresponding change occurs in *RR* length, then, with large *IQR*, this indicates that a motion artefact is present in the signal.

**Fig 3:**
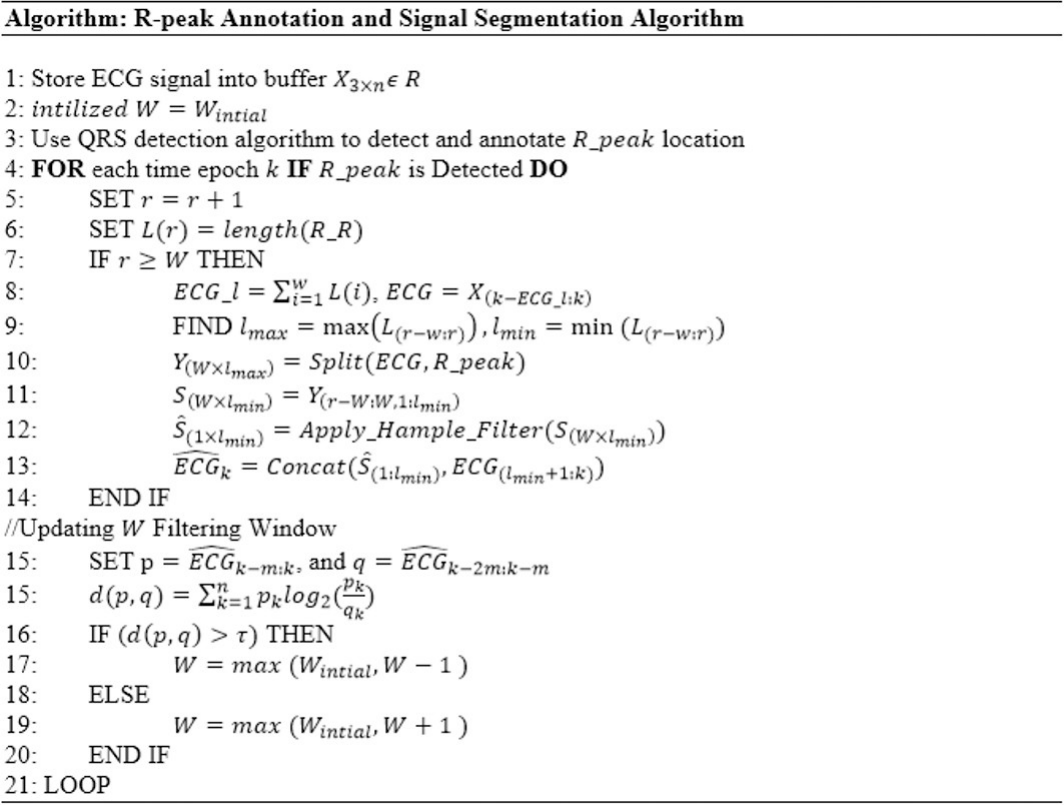
Hampel filter based estimation.

To capture the change in the ECG signal, the buffer size *w* should be updated online, accordingly. The use of relative entropy, also called the *Kullback*−*Leibler* divergence, is suggested to compare the probability density function of two windows that are taken from the filtered ECG signal. If the relative entropy is zero or near zero, then buffer size should not be decreased until the initial buffer size. Otherwise it should be in,creased until the entropy becomes a small value, as illustrated in Fig 3. For calculating the relative entropy, *p*(*x*) and *q*(*x*) are two probability distributions of a discrete random variable *x*. The relative entropy with respect to *q* can be obtained as follows:

$$d(p,q)=\sum_{k=1}^{n}p_{k}{log}_{2}(\frac{p_{k}}{q_{k}})$$ (12)

The duration of the signal can vary according to the heartbeat rate information. In this paper, *l* is estimated from the information stored in the *Buffer*_*k*(*l*,*w*)_ as follows. Since the minimum duration of a single P-QRS-T signal is around 0.14 seconds when the heartbeats rate reaches 300 per minute and the maximum duration of a single P-QRS-T around 1.2 seconds when heartbeat rate falls to 50 per minute [47], the maximum value of *l* is 0.14 and the minimum value is 1.2. Meanwhile, *l* is estimated by subtracting two successive QRS peaks. Although the buffer width *l* is adjusted automatically in this paper, the maximum and minimum values are used as a cutoff threshold to avoid taking a wrong P-QRS-T length. This values can be adjusted manually by the physician or the user based on information obtained through medical examination. For example, athletes may have heartbeat rate around 38, thus, the maximum value should be adjusted to be 1.58 seconds. In case of undetected QRS complex, the *l* takes the pervious value. The algorithm in Fig 3 summarizes the steps of the second stage ECG filtering which include: the R-peak Annotation and Signal Segmentation Algorithm and invoke the Hampel filter estimation algorithm. Meanwhile, Table 1 describes the symbols used in the algorithm.

**Table 1:** List of the symbols used.

Symbol	Description
*W*	Filtering Window Size
*r*	Index of detected *R*−*peak*
*L*(*r*)	Number of samples between the current *R*−*peak* and the preceding one
*k*	Current time index
*ECG*	Buffered of previous ECG signal
$Y_{\left(W\times l_{max}\right)}$	*W* ECG signals stored on *W* rows with length *l*_*max*_
*L*,*l*_*max*_, *l*_*min*_	*L* vector contains lengths of all *P*−*T* elements in the buffer, length, *l*_*max*_ is length of the *longest P*−*QRS*_−*T and l*_*min*_ *is the length of the shortest P*−*T*
${\hat{S}}_{\left(1\times l_{min}\right)}$, ${\hat{ECG}}_{k}$	Matrix of the estimated ECG signal samples
${\hat{ECG}}_{k-m:k},\;{\hat{ECG}}_{k-2m:k-m}$	The estimated signal during the time interval, *k*−*m* until *k*, where *m* is the integer that indicates the period of time the ECG is stationary.

### Hampel Filter-Based Estimation Module (HFBE)

The idea behind suggesting Hampel filter for removing motion artefacts in the ECG is that the successive ECG segments have a repetitive structure while the motion artefact is random in each segment. Thus, the Hampel filter can recover the original ECG structure from a noise contaminated one. The Hampel filter needs four important parameters to perform the estimation: the median ∅_*k*_, the median absolute deviation (MAD) *δ*_*k*_, Hampel filter upper bound *HUB*_*k*_, and Hampel filter lower bound *HLB*_*k*_. The Hampel filter’s parameters are calculated as follows.

$$Hample\;Filter\;Paramters\left\{ \begin{array}{c} \begin{array}{c} \emptyset_{k(i)}=median(S_{\left(k:k-w,l\right)})\\ \delta_{k(i)}=1.4826\times median\{\left|S_{\left(k:k-w,l\right)}-\emptyset_{k(i)}\right|\} \end{array}\\ \begin{array}{c} {HUB}_{k(i)}=\emptyset_{k(i)}+m\times \delta_{k(i)}\\ {HLB}_{k(i)}=\emptyset_{k(i)}-m\times \delta_{k(i)} \end{array} \end{array}\right.$$ (13)

where *k* is the time epoch of the complete ECG signal and *i* is one sample of the ECG segment, *w* the buffer size (in terms of number of the ECGs segments in the buffer), and *l* is the length of the ECG segment.

The filter response is given by:

$$h(S_{k(n)})={\hat{S}}_{k(n)}=\left\{ \begin{array}{l} S_{k(n)}\;{HLB}_{k(n)}\leq |S_{k(n)}|\leq {HUB}_{k(n)}\\ \;\emptyset_{k(n)}\;otherwise \end{array}\right.$$ (14)

Therefore, the estimated signal will be as follows:

$${\hat{S}}_{k}=[\begin{array}{ccc} {\hat{S}}_{k(1)} & {\hat{S}}_{k(2)} & \begin{array}{ccc} {\hat{S}}_{k(3)} & \cdots & {\hat{S}}_{k(N)} \end{array} \end{array}]$$

**Fig 4:**
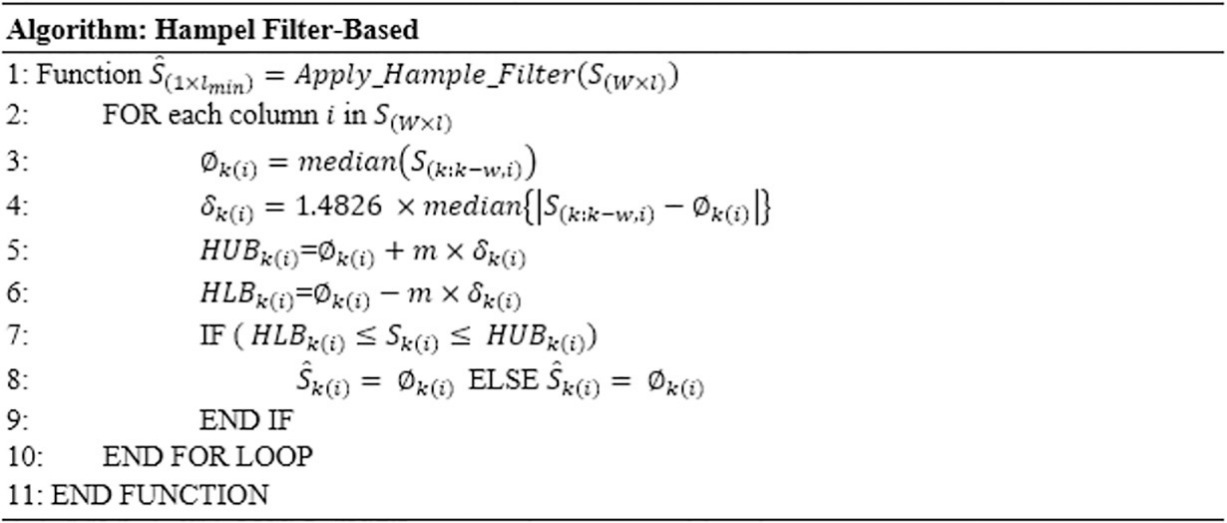
Hampel filter-based estimation.

Fig 4 shows a Hampel filter-based function that takes the buffer content as input and returns the filtered signal.

The input of the Hampel filter can be written as vector containing the complete ECG signal (P-QRS-T) that represents a cardiac cycle of the ECG signal as follows:

$$S_{k}=[S_{k-\frac{l}{2}},S_{k-\frac{l}{2}+1},\ldots S_{k},\ldots ,S_{k+\frac{l}{2}-1},S_{k+\frac{l}{2}}]$$ (15)

while the output of the Hampel filter, ${\hat{S}}_{k}$, is a vector resulting after applying the Hampel filter function in Fig 4. Recursive Hampel filter is performed based on replacing the sequence of the old filtered data by the output of the Hampel filter estimation sequence, as follows:

$${\hat{S}}_{k}=[{\hat{S}}_{k-\frac{l}{2}},{\hat{S}}_{k-\frac{l}{2}+1},\ldots {\hat{S}}_{k},\ldots ,{\hat{S}}_{k+\frac{l}{2}-1},{\hat{S}}_{k+\frac{l}{2}}]$$ (16)

**Fig 5:**
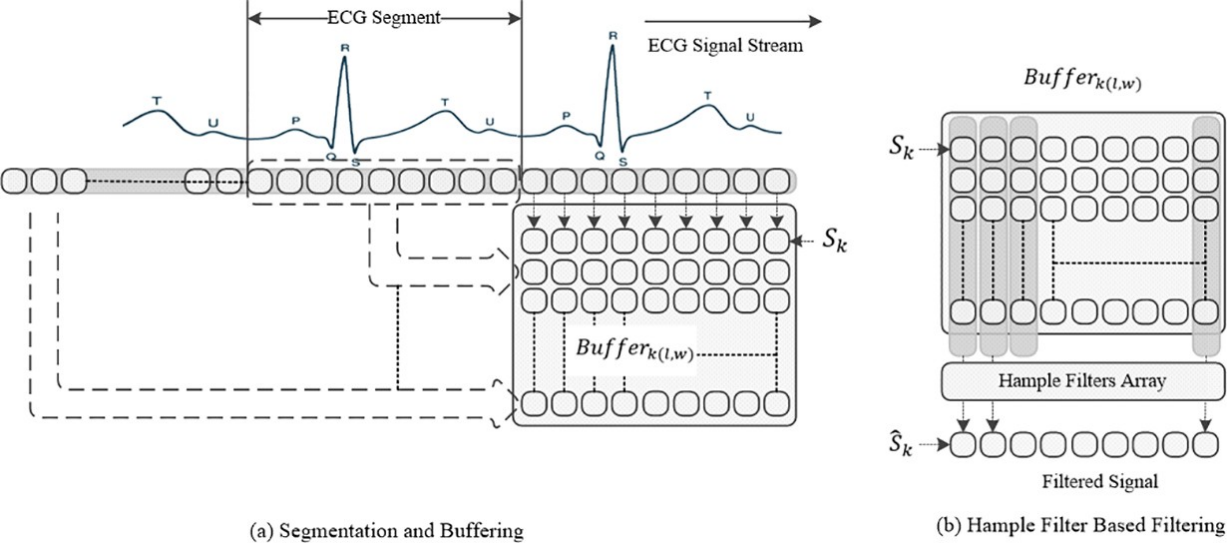
Conceptual structure of hampel filter-based filtering.

Fig 5 illustrates the conceptual structure of the proposed idea. The ECG segmentation and buffering are shown in Fig 5(A), while the Hampel-based filtering is shown in Fig 5(B).

## Performance evaluation

The effectiveness of the proposed algorithms was evaluated in the Matlab environment. The Mobile HEALTH (MHEALTH) dataset [11, 49] were used to evaluate the proposed algorithm. MHEALTH dataset comprises body motion and vital signals recordings from ten healthy subjects while performing daily activities (e.g., sitting, walking) and exercise activities (e.g., cycling, frontal elevation of arms, and Knees bending). The ECG electrodes were placed on the subjects’ chest (with 2-lead ECG), right wrist and left ankle and attached by using elastic straps while the acceleration sensors were placed on the central location on the chest near to the ECG electrodes. The proposed algorithm was evaluated using data collected from the ECG electrode that was placed on the chest. Similarly, the used acceleration data by the adaptive filter were collected form the acceleration sensors which was placed on the chest near to the ECG electrode. The information was recorded from ten healthy subjects while performing twelve different physical activities, as illustrated in Table 2. Shimmer2 [BUR10] wearable sensors were used for the recordings. All sensing modalities were recorded at a sampling rate of 50 Hz, which is considered sufficient for capturing human activity. MHEALTH dataset is found to generalize to common activities of the daily living such as sitting, walking and running, the intensity of the actions while performing the activities and their execution speed or dynamicity. The data were recorded in an out-of-lab environment with no constraints on the way these must be executed, with the exception that the subject should try their best when executing them. The datasets have been used for building many mobile-based health applications, such as in [12, 50]. MHEALTH is freely available online and can be downloaded from the following link “https://archive.ics.uci.edu/ml/datasets/MHEALTH+Dataset” which was last retrieved on 5-June 2018.

**Table 2:** MHEALTH datasets.

Code	Physical Activity	Duration	Code	Physical Activity	Duration
L1	Standing still	1 min	L7	Frontal elevation of arms	20X
L2	Sitting and relaxing	1 min	L8	Knees bending (crouching)	20X
L3	Lying down	1 min	L9	Cycling	1 min
L4	Walking	1 min	L10	Jogging	1 min
L5	Climbing stairs	1 min	L11	Running	1 min
L6	Waist bends forward	20X	L12	Jump front & back	20X

As the MHEALTH dataset contains different activities, the reference P-QRS-T segment can be used for the evaluation which can be obtained when subject’s activity is low, such as standing still, sitting and relaxing, and lying down. However, due to the non-stationary nature of the ECG signal recorded during relaxing, this cannot be used to evaluate the ECG signal during running or jogging. Therefore, a ground truth ECG reference signal is unavailable for the evaluation. In addition, the absence of a ground truth motion artefact noise reference makes it difficult to evaluate the effectiveness using signal to noise ratio (SNR). In addition, QRS complex detection is not a reasonable measure for evaluating motion artefact reduction methods because of the presence of highly accurate QRS detection methods that can accurately detect the QRS complex even if there is a motion artefact or poor ECG signals are used. Therefore, in order to evaluate the effectiveness of the proposed method, the morphological change in the ECG is recorded and evaluated.

### Experimental results and discussion

In this section, the results of the proposed filtering algorithms are presented and organized in three subsections as follows. In the first subsection, analysis of the correlation between the ECG and the acceleration signal is conducted. Then, the proposed weighted adaptive filtering method (WAF) is evaluated and compared to the conventional adaptive filter (AF) method. Finally, the Hampel based filtering algorithms (HFBE and the Recursive Hampel filter RHFBE) are evaluated and compared to the AF and WAF algorithms.

#### Analysis of the relationships between acceleration and motion artefact

**Fig 6:**
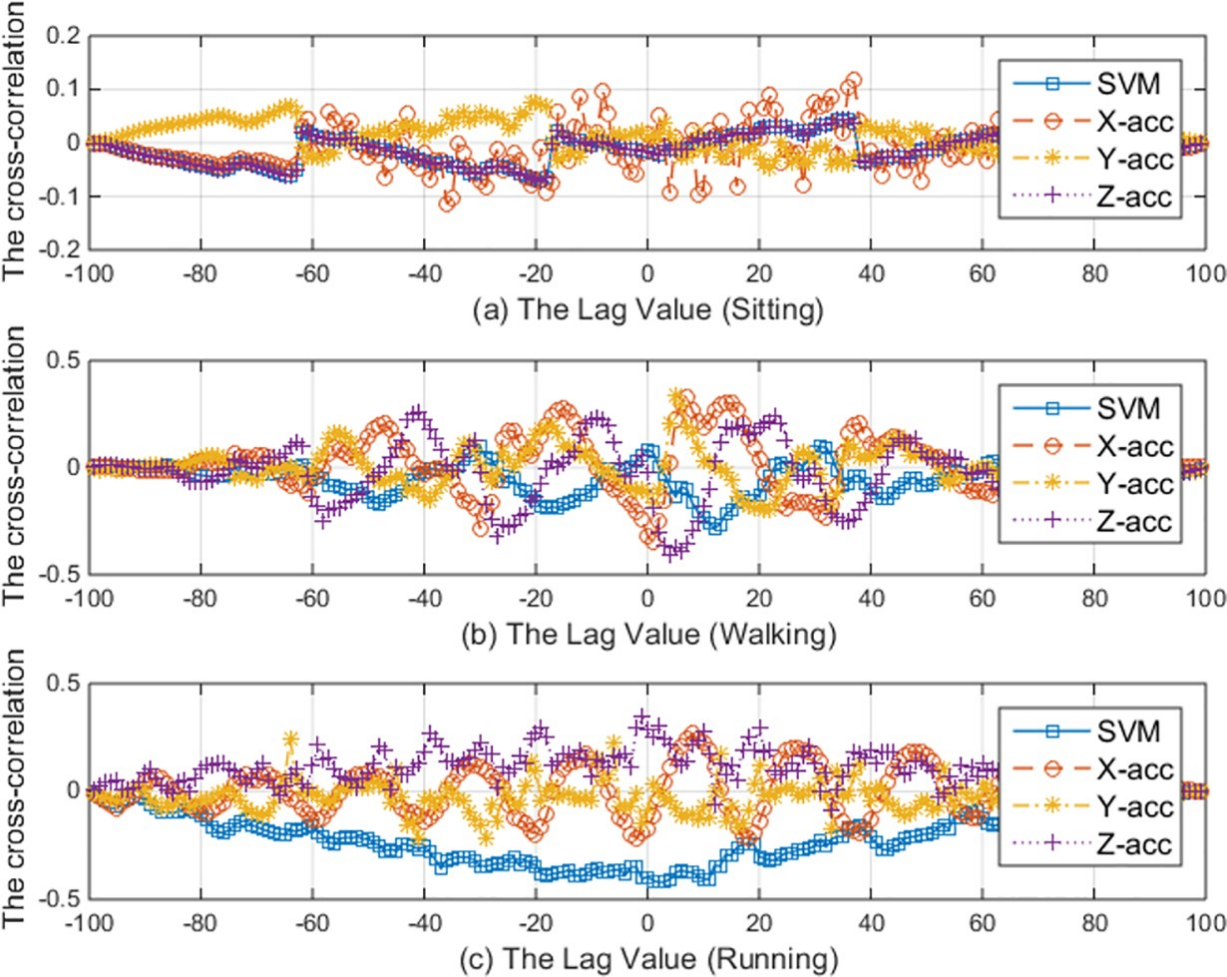
The correlation between the acceleration signals and the ECG signal at different lags.

In this section, the correlation between the motion artefact contaminated ECG signal and the acceleration signal has been studied. Fig 6 shows the correlation coefficient at different lags. Four acceleration signals were studied as follows, the acceleration in the longitudinal direction (X-acc), the acceleration in latitude direction (Y-acc), the acceleration in the altitude direction (Z-acc), and the absolute value of the acceleration vector (Signal Vector Magnitude or SVM for short). SVM is the absolute total value of the acceleration in all directions, so that has been used to summarize the correlation between the ECG and the acceleration [36, 37]. It can be noticed from Fig 6(A), 6(B) and 6(C) that the correlation slightly changes according to the activity types. For example, when the subject is sitting and relaxing the correlation is lower than 0.1 and, when the subject is walking or running the correlation insignificantly increases lower than 0.5. This low correlation makes the improvement gained by the adaptive filtering is not so promising if the acceleration signal is used as a reference in the adaptive filters.

**Fig 7:**
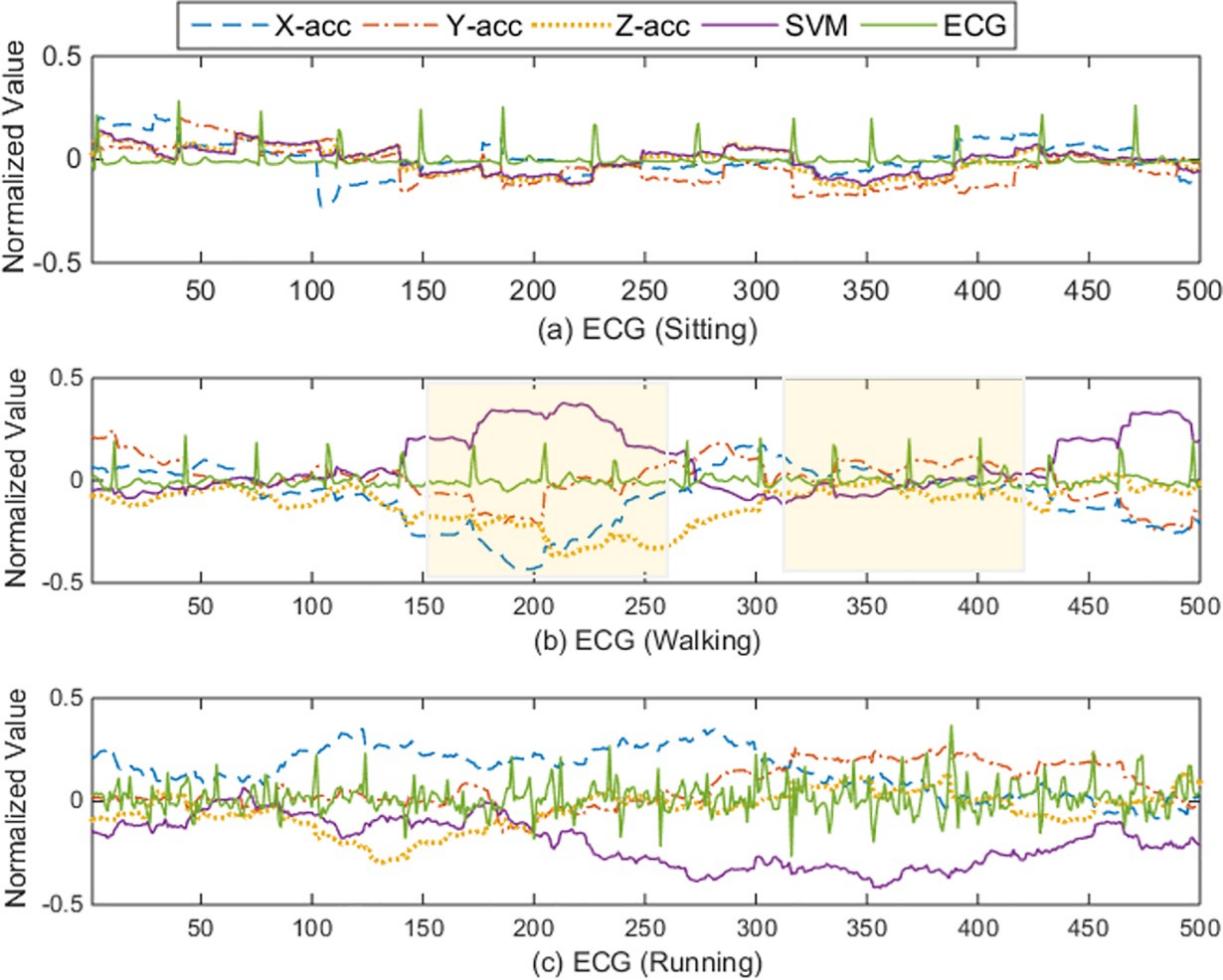
The correlation between the ECG signal and the acceleration signal.

To further study the correlation in more details, Fig 7 shows the detailed explanation of the correlation between the acceleration signals and the ECG signal at lag zero. The x-axis represents the sample index while the corresponding y-axis represents the normalized value of the ECG signal and the correlation coefficient between the raw ECG signal and the acceleration signals. It can be seen in Fig 7(A) that correlation is lower than 0.2 with the signals that were recorded while the person is sitting. Meanwhile, there is an occasional and low correlation among the signals that were recorded while the subject is walking or running as shown in Fig 7(B) and 7(C), respectively. By looking at the shaded areas in Fig 7(B), in the first shaded area from the left, the signal was slightly contaminated by motion artifact, and the correlation is around 0.4. Meanwhile, in the second shaded area, the correlation is about 0.1 with the ECG signal was slightly affected by the motion artifact. The similar random behavior of the correlation can be noticed in Fig 7(C). Based on this analysis, it can be concluded that the correlation between the acceleration signal and the ECG signal is weak and not necessarily linear, it occurs occasionally and randomly according to the electrode displacement status from the subject skin. Therefore, it is expected that the adaptive filter with the acceleration signal as the reference will not adequately improve the quality of the motion artifact contaminated signal. More seriously, due to the random correlation between the ECG and the acceleration signal, the conventional adaptive filtering may introduce more noise in the signal due to this random correlation. The detailed performance evaluation of the adaptive filtering with the acceleration signal as a reference has been discussed in the next section.

#### Evaluating the weighted adaptive filter method

**Fig 8:**
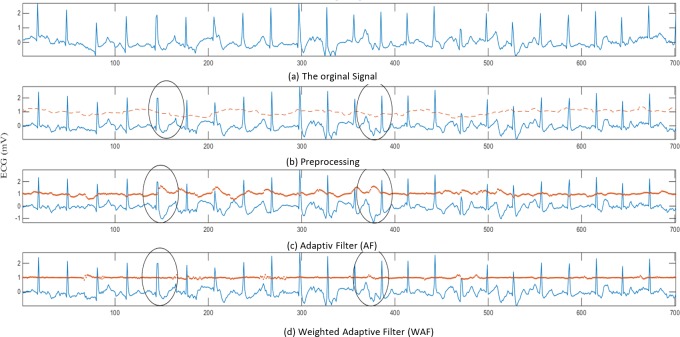
Filtering results using the ECG signal recorded during walking.

**Fig 9:**
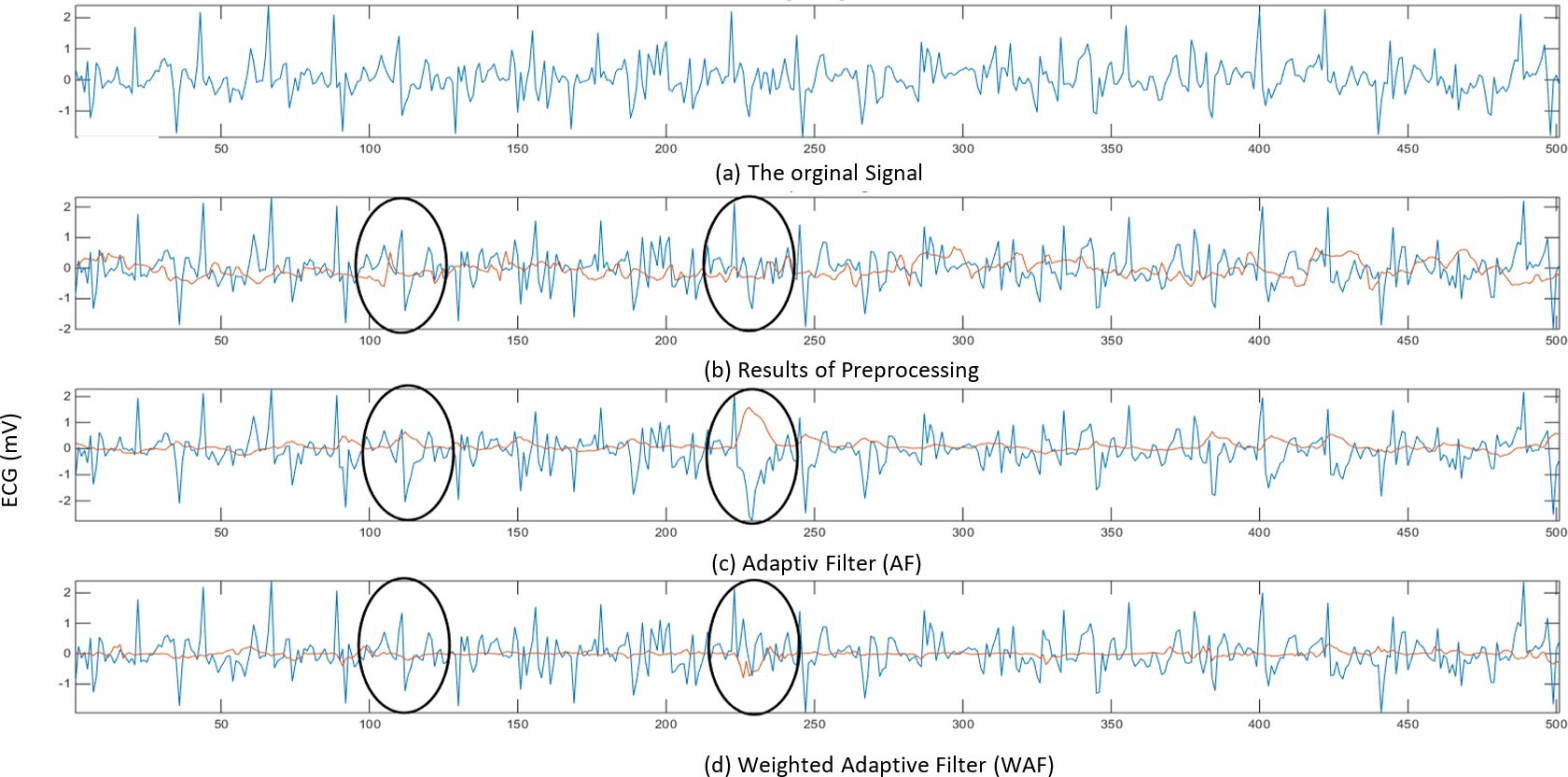
Filtering results using the ECG signal recorded during running.

In this section, the effectiveness of the adaptive filtering methods is studied. The results obtained by applying the proposed weighted adaptive filter to different ECG signal recorded while the subject performing different activities are discussed and compared to the conventional adaptive filter. Figs 8 and 9(A), 9(B), 9(C) and 9(D) shows comparison between the adaptive filter (AF) with the proposed weighted adaptive filter (WAF) while the subject performing two types of activities, walking, in Fig 6, and running, in Fig 9. The raw ECG signal is shown in Figs 8 and 9(A), the output of the pre-processing is shown in Figs 8 and 9(B), while the output of the conventional adaptive noise filter (AF) is shown in Figs 8 and 9(C) and the output of the proposed filter (WAF) is shown in Figs 8 and 9(D). As shown in Figs 8 and 9(A), the raw ECG signal is very noisy, due to the presence of the motion artefact in the signal. Due to the overlapping nature of the motion artefact, it is still present in the ECG presented in Figs 8 and 9(B), even after pre-processing using conventional digital filtering of noise and the adaptive baseline noise cancelling (AF). As illestrated in Figs 8 and 9(C), the results of the adaptive noise filter with acceleration as a reference show that there is no sign of enhancement, due to the weak correlation between the noise and the ECG. Moreover, the weak correlation of the noise and the ECG leads to wrongly adapt the filter’s coefficient and thus produce incorrect estimations of the motion artefact. Accordingly, the acceleration signal misleads the adaptive noise filter and thus produces the wrong filtering output. This is clear when comparing the circles in Figs 8 and 9(B) with the circles in Figs 8 and 9(C). In contrast, the distortion of the signal is reduced using the proposed Weighted Adaptive Noise Filter (WAF), as shown in the circles in Figs 8 and 9(D). The cause behind the distortion in the ECG signal is that the acceleration signal has low correlation with the noise in the ECG and higher correlation with ECG signal itself. This prevents the adaptive filter from reaching optimal convergence, and thus poor estimation of the motion artefact.

To quantitatively evaluate the performance of the adaptive filter algorithms regarding the motion artefact reduction, the power ratio (PR) is used as an indication for the improvement of the quality of the motion artifact contaminated ECG signals [42, 51]. PR is the ratio between the power of the primary input signal (the motion artifact contaminated signal) and that of the desired signal (the output signal of the adaptive filter) and it can be expressed as follows.

$$PR=\frac{E({\left|Ps_{ecg}\right|}^{2})}{E({\left|{Pd}_{ecg}\right|}^{2})}$$ (17)

Where *E*(|*Ps*_*ecg*_|^2^) represents the power value of the raw ECG signal before the filter, *E*(|*Pd*_*ecg*_|^2^) is the power value of the desired signal (after filteration). In adaptive filter, the error signal is the desired information i.e. the filtered ECG signal. Table 3 shows the power ratio of both the proposed weighted adaptive filter algorithm (WAF) and the conventional Adaptive Filter (AF). In most cases, PR_WAF is higher than PR_AF because the WAF modifies the original signal according to the correlation coefficient value. Meanwhile, AF continuously adjusts the signal even if their signal is not affected by the body movement. It is clear from Figs 8 and 9 when the segments presented in the oval shapes are compared. It can also be noticed in Table 3 in which the power ratio of AF (PR-AF) is lower than one due to the random correlation between the noise and the ECG which had lead AF to make an unnecessary modification to the ECG signal. Also, it can be noted from Table 3 that the PR of the proposed algorithm WAF is almost one because of the weak correlation between the noise and the acceleration signal. This weak correlation has led the proposed algorithm WAF to keep the original signal from unnecessary modification (see also the segments presented in the oval shapes in Figs 8 and 9). This result suggests that the WAF is more effective than the AF filter in case of random correlation between acceleration and the ECG signal. At least the proposed weighted adaptive filter WAF preserves the original signal from being destoried through the random corelation between the motion artefact and the acceleration, while the conventional adaptive filter AF wrongly modifies the ECG signal. Consequently, the AF filter may lead to false analysis of the cardiac.

**Table 3:** Performance statistics of the adaptive filtering algorithms at different activity levels.

Activity Level\Adaptive Filter	AF	WAF
Standing still	0.83	0.98
Sitting and relaxing	0.88	1.00
Lying down	0.88	1.01
Walking	0.84	0.99
Climbing stairs	0.80	0.99
Waist bends forward	0.87	1.00
Frontal elevation of arms	0.75	1.00
Knees bending (crouching)	0.83	1.00
Cycling	0.82	0.99
Jogging	0.87	1.00
Running	0.78	0.99
Jump front & back	0.83	0.98
Average	0.83	1.00
Standard Division	0.04	0.01

In contrast to as the studies presented in [3, 4, 19, 40] which used the acceleration signal as a reference for adaptive noise cancelling, it can be concluded that using the acceleration signal as a reference for the adaptive filtering algorithms can not impressively improve the quality of the motion artifact contaminated signals in the ECG signals recorded during normal activities using wearable ECG sensor. This fact has also been noticed by Zhang et al. in [42]. However, the author suggested that the failer of the adaptive filtering algorithm to improve the quality of the motion artifact contaminated signal refers to the position of the acceleration sensor. It was recommended that the acceleration sensors should be mounted in the ECG electrodes to capture the electrode movement. Although the acceleration sensors have been integrated into the ECG electrodes in MHEALTH datasets, the improvement still low due to the low correlation between the acceleration signal and the motion artifact portion in the ECG signal. The low correlation is due to that the motion artifact in the ECG signal is mainly generated from the expansion of the skin under the electrode which causes a change in the potential thus the noise is created. Meanwhile, the acceleration signal is related to the physical movement, and it has nothing to do with the possibility. That is, the noise signal and the acceleration signal are generated from different mechanisms which are not highly correlated [19–21, 42]. The correlation is reliable only if the movement leads to displace or slide the electrode on the skin. Besides, the physical movement is not necessarily always lead to supplant of the ECG electrode if the electrode has been mounted well on the skin surface. Therefore, the adaptive filter which uses the acceleration as a reference can not effectively improve the quality of the motion artifact contaminated ECG signals. This finding confirms our previous hypothesis in [52]. Next section evaluate the performance of the proposed Hample filter based estimation.

#### Evaluating Hampel-Based filtering (HFBE and RHFBE)

**Fig 10:**
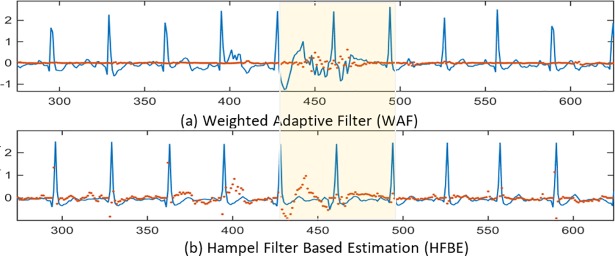
Filtering results using the ECG signal recorded during free movement.

In this section, the effectiveness of Hampel-based filtering is evaluated. The performance of the both HFBE and RHFBE are studied and compared. Fig 10 gives quick insights into the effectiveness of the Hampel-based filtering concept.

In Fig 10(A) and 10(B), the *x*-axis represents the time epochs while the *y*-axis represents the ECG potential intensity. The solid line represents the ECG signal after applying the Hampel based filter. Meanwhile, the dotted line represents the removed motion artefact signal which is the difference between the signal before and after filtering (the removed portion from the original signal). The highlighted region in the Fig 10 represents the portion of the signal that has a motion artefact. Fig 10(A) illustrates the output filtered ECG signal using the proposed WAF method, as well as the filtering residual (drawn as dots). As shown in the Fig, the residual of the WAF is almost zero, which implies that the correlation between the motion noise reference and ECG recording is very weak. Thus, WANC keeps the original signal without further destruction. In Fig 10(B), the results of applying the HFBE method are presented. As shown in the Fig 10, HFBE is effective for eliminating motion artefact.

**Fig 11:**
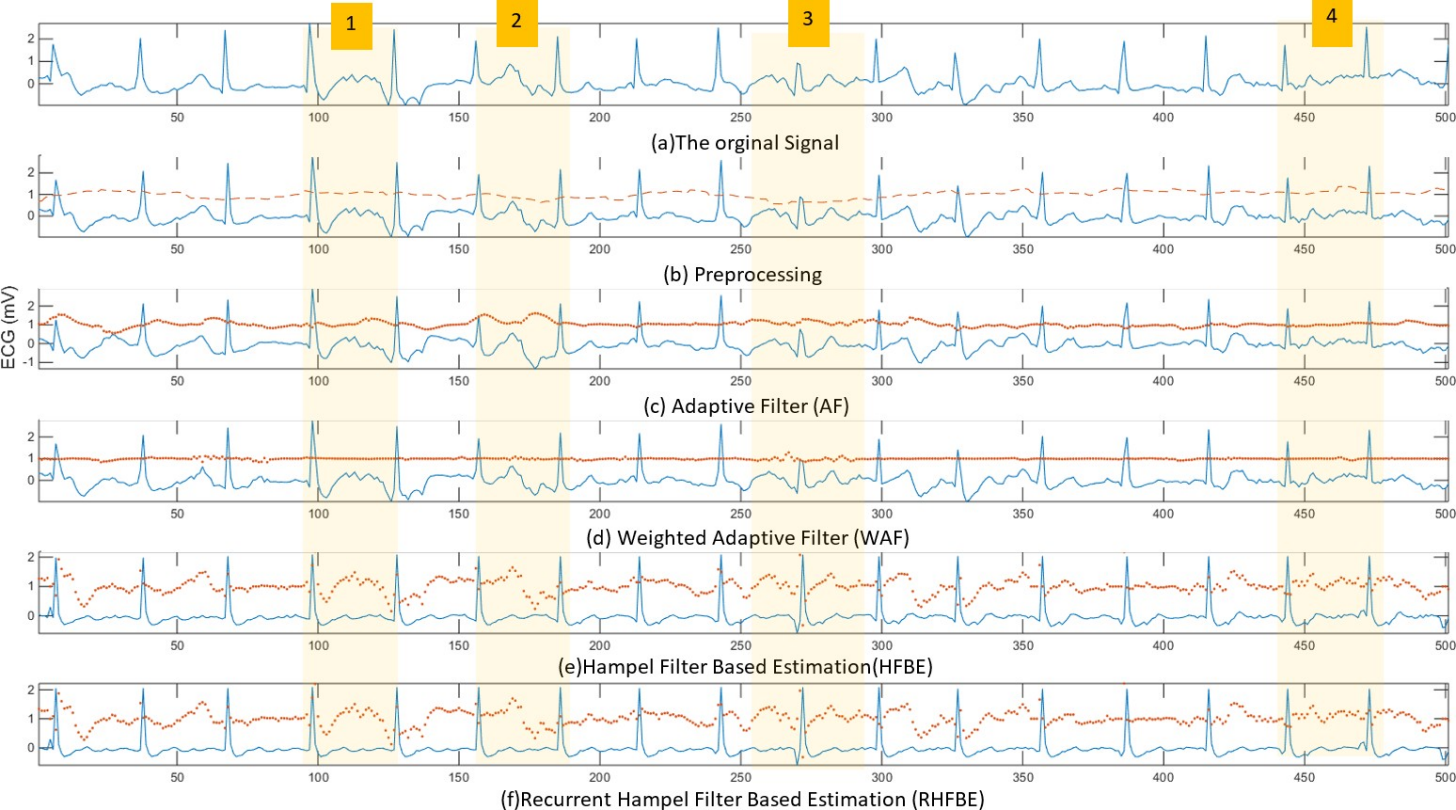
Filtering results using the ECG signal recorded during knee bending exercise.

Fig 11(A), 11(B), 11(C), 11(D) and 11(E) illustrate five ECG signals, as follows. Fig 11(A) shows the raw ECG signal extracted from subject number 2 during knee bending exercise. Fig 11(B) represents the ECG signal after the pre-processing stage. It also shows the real-time correlation coefficient between the ECG and the acceleration information. Fig 11(C) shows the output of the adaptive noise cancelling algorithm (AF) after pre-processing and the acceleration signal used as a reference. It also includes the estimation of the motion artefact noise that is generated using the AF algorithm. Fig 11(D) shows the desired ECG signal after first stage filtering using the proposed filtering WAF algorithm which works based on the correlation between the motion artefact noise reference and the contaminated ECG signal. Fig 11(E) illustrates the output of the second filtering stage, namely using the HFBE algorithm. Finally, the results of the recursive HFBE are presented in Fig 11(F).

**Fig 12:**
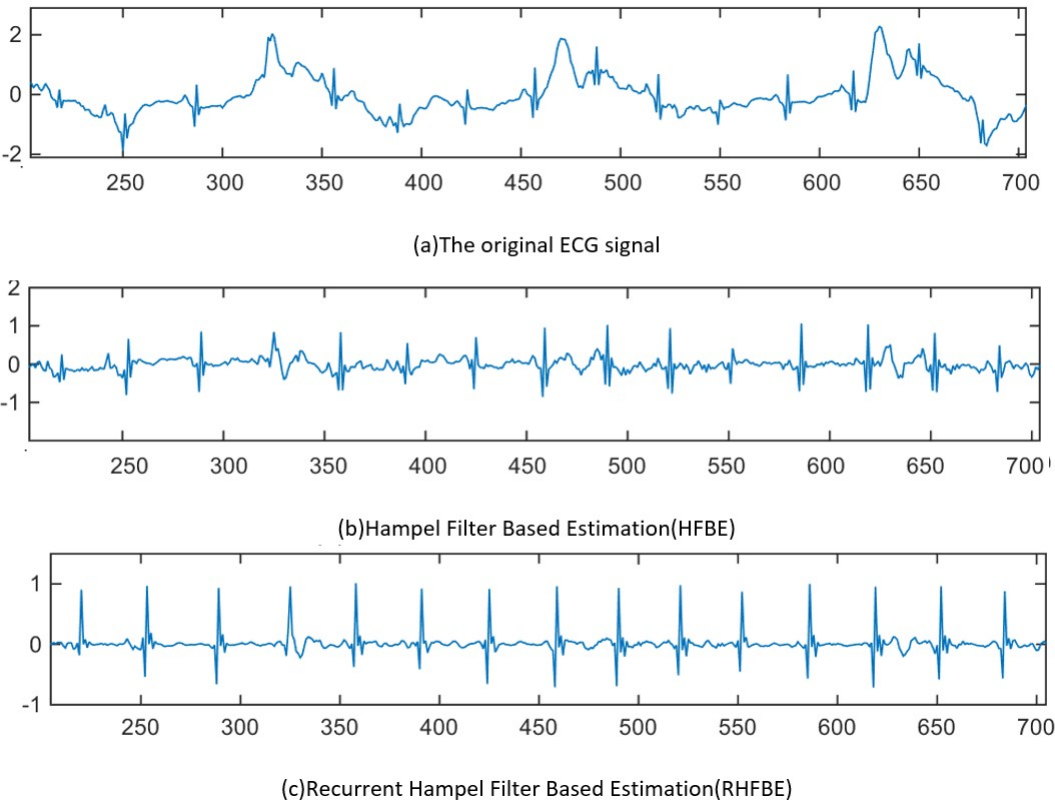
Example of treatment of heavey contaminated ECG signal.

To simplify the explanation, four regions in Fig 11 are highlighted. In the first region, the P and T wave positions are highlighted. As can be seen in Fig 11(A), the P and T wave’s are both contaminated by the motion artefact that is appearing as an atrial fibrillation disorder. As shown in Fig 11(C) and 11(D), neither ANC nor WAF could remove the motion artefact. However, both of the Hampel filter-based estimation algorithms (i.e. HFBE and RHFBE) show their effectiveness in reducing the impact of motion artefacts on the ECG signal, as shown in Fig 11(E) and 11(F). RHFBE motion artefact noise removal performs better than HFBE in terms of better showing the structure of the ECG signal, as shown in Fig 11(F). Similarly, as shown in Fig 11(A) and 11(B) in the second highlighted region, P and T-waves are corrupted by different types of motion artefact. Again, neither of the adaptive filters (ANC and the proposed WAF could remove the motion artefact noise, due to the weak correlation with the acceleration signal. As can be observed in Fig 11(C) and 11(D), ANC has wrongly modified the morphology of the P and T-waves while WAF retains the original noise. In contrast, both HFBE and RHFBE have significantly reduced the impact of the motion artefact on the ECG signal, as can be seen in Fig 11(E) and 11(F). In the third highlighted section, the QRS complex is distorted but it is perfectly recovered by both HFBE and RHFBE. In the same way, the disturbed P and T waves in the fourth highlighted region are recovered by HFBE and RHFBE. Fig 12 shows the performance of the Hampel filter based estimation on massive motion artefact contaminated ECG signal. It is clear that the Hambel filter based estimation can accurately improve the quality of the ECG signal even under high noise.

**Fig 13:**
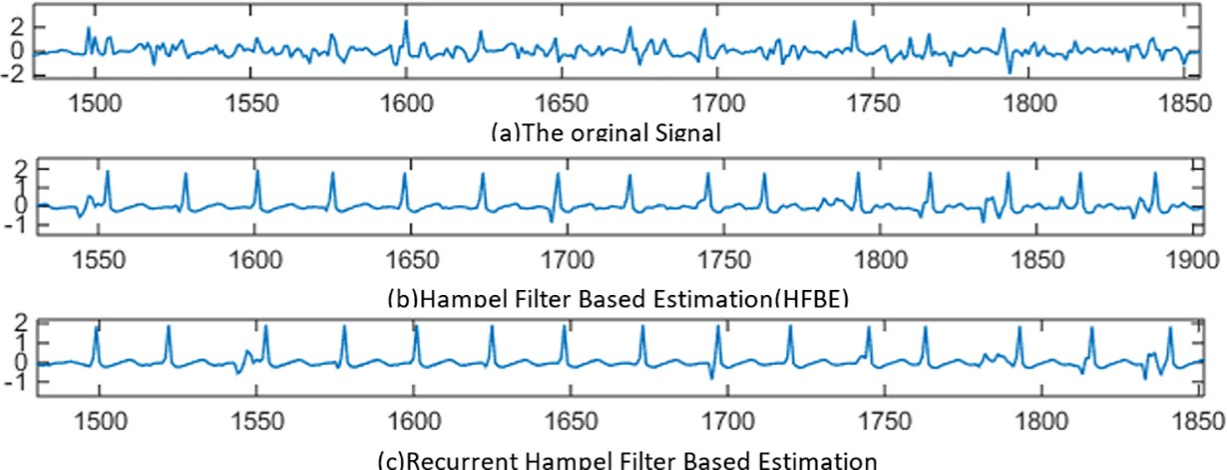
Filtering results using the ECG signal recorded during walking following by jogging exercise.

Table 4 shows the percentage of the removed motion artefact from the contaminated P-QRS-T segments for the raw ECG records and after the treatment by the adaptive filters and the proposed filters (WAF, HBE, and RHFBE). If any part of the P-QRS-T is corrupted then it is counted as motion artefact contaminated segment. The motion artifact are manually checked by an expert before and after the application of the tested filtering algorithms. The results show that the amount of motion artefact differ based on the type of the activity performed by the subject. For example, during the lying down or sitting and relaxing the percentage of the motion arefact is lower than 15% while it has increased to 100% during Jogging and running. In terms of the percentage of motion artefact that have been removed by the tested algorithms, the conventional adaptive filter fails to remove the motion artefact from many signals. More seriously, AF has introduced another noise to the original ECG signal (see the negative sign). Meanwhile, WAF has removed low percentage of the motion artefact while, it fails to remove large portion of the motion artifact. The average achievement by the WAF is around 23±20%. This low achievement is due to the low direct correlation between the motion artifact signal and the acceleration signal. Both Hampel filter based estimation algorithms have performed well with many activities, especially when the raw ECG signal is partially contaminated by the motion artifact. For example, if the motion artifact around 15% of the total signal then HFBE and RHFBE archives 100% removing of the motion artefact. However, with some activities even if the percentage of the motion artefact is low such as in Knee bending and Jump front and back exercise (less than 20%), the improvement still not high (82%). This because the motion artefact affect many subsequent P-QRS-T segments. It can be noticed from the table, Hampel filter based estimation fails to remove the motion artefact during jogging and running exercise. This is due to mean reasons as follows. The first, the performance of the QRS detection algorithm during the heavy exercise and the second is the fact that the majority of the subsequent P-QRS-T segments are contaminated by the noise. The reason behind decreasing of the QRS detection could be due to the low quality of the recording ECG equipment. It may not be attached well to the subject body. Fig 13(A) shows example of heavy contaminated ECG signal which was recorded during jogging exercise. The second reason is due to that the majority of the subsequent ECG segments are contaminated by the noise (as shown in Fig 13(A)). However, The proposed Hampel filter based algorithm can still improve such signal when the subject move from one activity to another such as from walking to jogging (as shown in Fig 13(B) and 13(C). It can be concluded that Hampel filter based estimation is effective only for the signals which are partially contaminated by the motion artefact that is the majority of the P-QRS-T segments are partially affected by the noise as shown in Fig 12.

**Table 4:** Percentage of the removed motion artefact from the raw signal.

Activity	Artefact Percentage in the raw signal (%) (BEFORE)	Percentage of Removed Motion Artefact (%)(AFTER)			
		AF	WAF	HFBE	RHFBE
Standing still	9.68	-11.11	22.22	100	100
Sitting and relaxing	10.26	-50.00	25.00	100	100
Lying down	15.00	-33.33	0.00	100	100
Walking	14.81	-66.67	61.11	88.89	94.44
Climbing stairs	57.78	25.00	40.38	88.46	84.62
Waist bends forward	18.75	5.56	50.00	88.89	88.89
Frontal elevation of arms	41.67	2.50	37.50	90.00	85.00
Knees bending (crouching)	20.24	-11.76	5.88	82.35	82.35
Cycling	34.03	-22.45	28.57	85.71	91.84
Jogging	100.00	0.00	0.00	12.18	7.69
Running	100.00	0.00	0.00	13.33	8.67
Jump front & back	20.24	-11.76	5.88	82.35	82.35
**Average**		**-14.50**	**23.05**	**77.68**	**77.15**
**Standard Deviation**		**24.32**	**20.21**	**29.65**	**31.50**

Table 5 shows the filtering performances, in terms of the power ratio, for the proposed algorithms (WAF,HF, and RHF) and the adaptive filter (AF). As stated earlier, the PR of the adaptive filter is lower than one which indicate that the AF has increased the power of the original signal due to the wrong adjustment of the adaptive filter parameters. Meanwhile, the proposed WAF keep the power of the original signal due to the influence of the proposed weights on the adaptive filter parameters. In case of Hampel filter based estimation, it can be noticed that the power ratios are higher than one because of the removing of the noise signal from the original ECG signal. Both HFBE and RHFBE perform well in terms of the power ratio improvement. However, RHFBE has removed higher portion of the original signal comparing with the power removed by the HFBE.

**Table 5:** Performance statistics of the tested algorithms at different activity levels.

Activity	AF	WAF	HFBE	RHFBE
Standing still	0.83	0.98	1.23	1.19
Sitting and relaxing	0.88	1.00	1.13	1.15
Lying down	0.88	1.01	1.08	1.06
Walking	0.84	0.99	1.09	1.06
Climbing stairs	0.80	0.99	1.24	1.24
Waist bends forward	0.87	1.00	1.11	1.09
Frontal elevation of arms	0.75	1.00	1.30	1.36
Knees bending (crouching)	0.83	1.00	1.21	1.24
Cycling	0.82	0.99	1.40	1.42
Jogging	0.87	1.00	1.20	1.21
Running	0.78	0.99	1.54	1.79
Jump front & back	0.83	0.98	1.23	1.19
Average	0.83	1.00	1.23	1.25
Standard Division	0.04	0.01	0.14	0.20

The performance of the proposed filtering methods was evaluated using QRS complex detection algorithms namely the Pan and Tompkins algorithm. Pan and Tompkins algorithm was proved to be the more effective compared with the state of the art R-peak detection algorithms [47]. The QRS detection results consist of the true positive (TP), false positive (FP), and false negative (FN). TP represents the total number of the QRS complexes that have been correctly detected. FP represents the total number of times the algorithm has wrongly detected QRS complex. FN represents the total number of the QRS complex that has not been detected. Three performance metrics have been used to quantify the improvement gain in the ECG signal namely, the sensitivity (SN), positive predictivity (PP), and the harmonic mean F-measure as shown in Eqs (18), (19) and (20), respectively. SN represents the percentage of the correctly detected QRS complex (heartbeats or the true positive rate or recall), while PP represents the percentage of detected heartbeats that are actually true (precision). F-measure is the harmonic mean which is used to measure the overall performance in the unbalanced datasets such as in the ECG dataset where the number of positive instances is (the R-peak) less than the negative instances. Table 5 shows the statistical results of the filtering performance in terms of the sensitivity (SN), positive predictivity (PP), and the harmonic mean F-measure. The results in Table 6 were obtained with respect to applying the filtering algorithms on an annotated copy of the MHEALTH dataset. The annotation is firstly performed using Pan and Tompkins QRS detection algorithm [46], and then it has been manually corrected by a medical expert.

**Table 6:** Statistical results of the filtering performance in terms of R-beak detection.

Activity	Raw ECG Signal					AF					WAF					HFBE					RHFBE				
	FP	FN	SN	PP	FM	FPR	FNR	SN	PP	FM	FPR	FNR	SN	PP	FM	FPR	FNR	SN	PP	FM	FPR	FNR	SN	PP	FM
Random Movement	75	2	98.0	56.7	71.8	8	2	98.0	92.5	95.2	8	2	98.0	92.5	95.2	7	2	100	93.5	96.6	6	2	100	94.3	97.1
Standing still	74	0	100	57.0	72.6	9	3	96.9	91.4	94.1	8	0	100	92.5	96.1	7	0	100	93.3	96.5	7	1	99	93.3	96.0
Sitting and relaxing	69	0	100	51.8	68.2	9	0	100	89.2	94.3	12	0	100	86.1	92.5	11	0	100	87.1	93.1	10	0	100	88.1	93.7
Lying down	52	0	100	58.1	73.5	3	1	98.6	96.0	97.3	4	0	100	94.7	97.3	3	0	100	96.0	98.0	3	1	98.6	96.0	97.3
Walking	86	0	100	48.5	65.3	18	0	100	81.8	90.0	20	0	100.0	80.2	89.0	19	0	100	81.0	89.5	18	0	100	81.8	90.0
Climbing stairs	107	0	100	49.1	65.8	11	0	100	90.4	94.9	11	0	100.0	90.4	94.9	10	0	100	91.2	95.4	9	0	100	92.0	95.8
Waist bends forward	79	0	100	49.0	65.8	16	0	100	82.6	90.5	16	0	100.0	82.6	90.5	15	0	100	83.5	91.0	15	1	98.7	83.3	90.4
Frontal elevation of arms	76	0	100	50.0	66.7	12	0	100	86.4	92.7	14	0	100.0	84.4	91.6	13	0	100	85.4	92.1	13	1	98.7	85.2	91.5
Knees bending (crouching)	94	0	100	47.8	64.7	12	0	100	87.8	93.5	12	0	100.0	87.8	93.5	11	0	100	88.7	94.0	11	1	98.8	88.5	93.4
Cycling	128	0	100	40.2	57.3	45	1	98.8	65.4	78.7	45	0	100.0	65.7	79.3	44	0	100	66.2	79.6	43	0	100	66.7	80.0
Jogging	125	17	75.7	29.8	42.7	88	23	64.3	33.8	44.3	82	15	77.1	39.7	52.4	82	24	69.8	40.0	50.9	87	23	70.0	42.0	52.5
Running	139	23	72.9	30.9	43.4	85	20	74.1	42.6	54.1	85	19	75.3	43.0	54.7	95	27	67.1	45.9	54.5	84	28	65.9	46.0	54.2
Jump front & back	40	1	96.2	38.5	55.0	20	0	100	56.5	72.2	20	0	100.0	60.5	75.4	17	0	100	59.1	74.3	17	0	100	60.5	75.4
**Average**	**88.00**	**3.31**	**95.60**	**46.72**	**62.52**	**25.85**	**4.00**	**94.67**	**76.65**	**83.98**	**25.92**	**2.77**	**96.18**	**76.93**	**84.80**	**25.69**	**4.08**	**95.15**	**77.76**	**85.04**	**24.85**	**4.46**	**94.59**	**78.28**	**85.18**
**Stander Deviation**	**29.61**	**7.53**	**9.54**	**9.28**	**10.14**	**28.77**	**7.85**	**11.52**	**20.41**	**17.10**	**27.45**	**6.39**	**8.89**	**18.74**	**15.27**	**29.72**	**9.54**	**11.86**	**18.78**	**15.90**	**28.65**	**9.41**	**11.87**	**18.44**	**15.54**

$$SN=\frac{TP}{TP+FN}$$ (18)

$$PP=\frac{TP}{TP+PN}$$ (19)

$$F-Measure=\frac{2*SN*PP}{SN+PP}$$ (20)

As illustrated in Table 6, concerning all tested filters, the QRS detection algorithm can accurately detect the R-peak of the recorded ECG signals during different activities. Although the results of applying the QRS detection algorithm on the raw ECG data reveals low performance during some harsh excercise, it performed well after preprocessing. It can be seen that all the proposed algorithms outperform the conventional adaptive filtering. On average, the overall performance, regarding F-measure, of the proposed algorithms is around 85%±15% while the adaptive filter AF archives 84±17. It can be seen that the proposed Hampel filter based estimators namely HFBE and RHFBE performed better as compared to the adaptive filters. Although the QRS detection algorithm can accurately detect the R-peak, it fails when the motion artifact wholly corrupts the QRS complex patterns. The performance of all algorithms, regarding F-measure, has been dropped to a low value during running and jogging exercises. The overall performance of all tested filters drops to 50%. This is because the motion artefact contaminates all the segments of the recorded ECG signals. Hampel filter based estimation assumes that the majority of the neighboring QRS complex is accurate. However, when the incoming subsequent P-QRS-T segments are corrupted for a long time, the Hampel filter based algorithm will not be useful. In most cases, the performance of the R-Peak detection algorithm is better with the proposed Hampel filter algorithm in terms of correctly identifies the QRS locations and the reduction of the false positive even during noises. It should be noticed that the evaluation concerning QRS detection accuracy is partially evaluated the performance of the motion artefact reduction algorithms. The reason is that this evaluation focuses on the QRS complex portions of the ECG segments while the remaining portion will not be evaluated.

**Fig 14:**
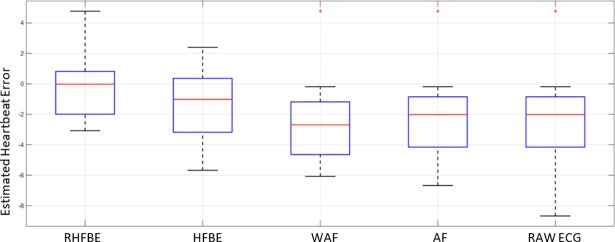
Summary of the performance during different forms of exercise.

Moreover, the performance of the proposed algorithms is further evaluated in terms of heartbeat rate estimation. Fig 14 illustrates the overall performance of the proposed methods in terms of heartbeat rate estimation error. Human estimation is used as ground truth knowledge, while QRS-based estimation is used as automatic estimation. The difference between human estimation and the QRS-based estimation is used as performance indicator. Heartbeat rate (HBR) is estimated during each activity as follows:

$$Estimated\;Heart\;Beat\;Rate\;\hat{\left(HBR\right)}=\frac{Number\;of\;Detected\;QRS}{Activity\;Duration\;(s)}$$ (21)

Meanwhile, the heartbeat rate estimation error (HBRE) is computed by subtracting the actual human-based estimation from the estimated heartbeat rate, $\hat{HBR}$, using the QRS complex algorithm.

As shown in Fig 14, each box plot summarizes the performance of one method applied on 12 different physical activities. When the median is near zero and the inter-quartile range (IQR) is small, the accuracy and precision of the method is better. Thus, RHFBE and HFBE methods show better performance than the adapted filter-based methods WANF, and ANF. The heartbeat rate error median is zero for the RHBE while it is near to minus one in HFBE. In addition, the IQR of the RHFBE is smaller than that of HFBE. Therefore, RHFBE has achieved a better performance than the other proposed methods.

**Fig 15:**
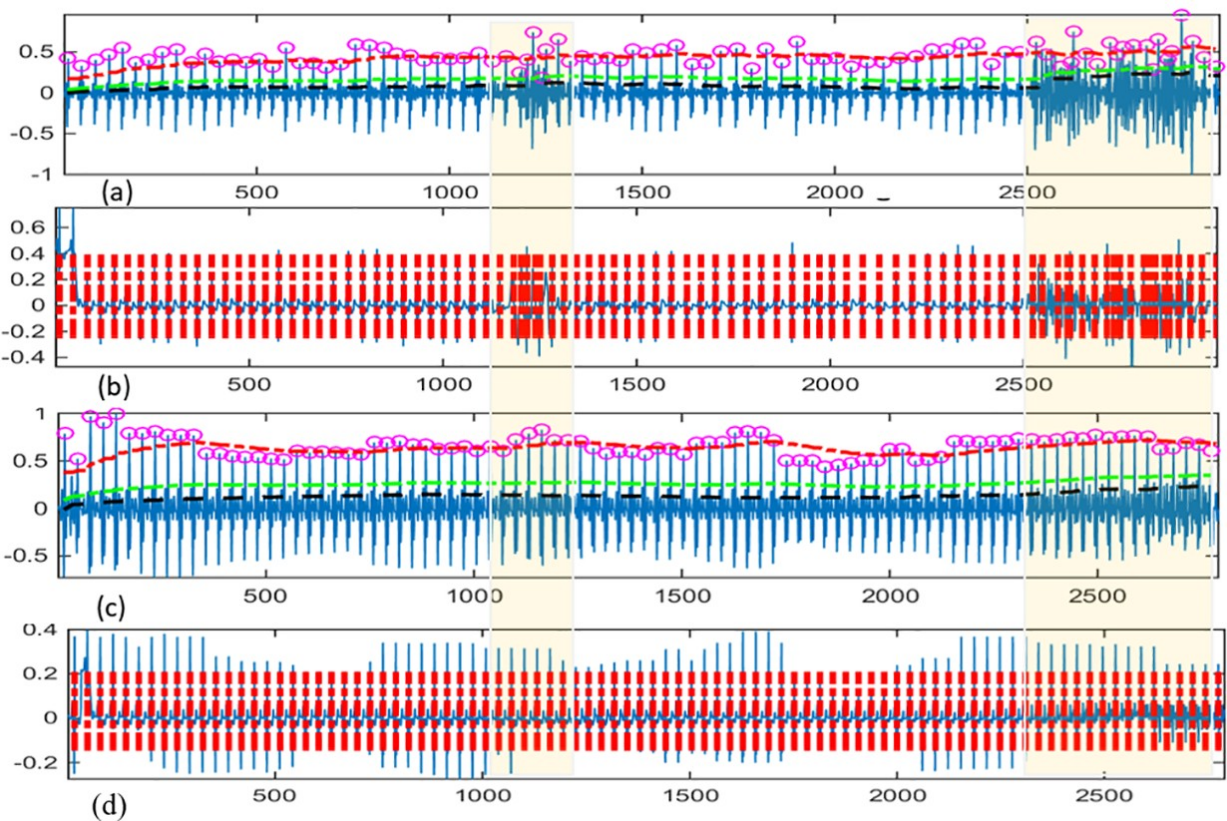
The QRS complex detection accuracy before and after the filtering using the proposed methods.

Fig 15 shows a detailed analysis of the QRS detection based evaluation. In Fig 15(A) and 15(B), the QRS detection algorithm is applied to the raw data. The small circles in Fig 15(A) and 15(C), and the corresponding heavy intermittent vertical lines in Fig 15(B) and 15(D) represent the position of the R-peak in the QRS complex which is detected using Pan and Tompkins QRS complex detection method. Fig 15(C) and 15(D) show the results of the application of the QRS detection algorithm on the filtered signal, i.e., after applying the proposed Hampel filter-based estimation method, namely HFBE and RHFBE. The heavy intermittent horizontal lines in Fig 15(A) and 15(C) are the threshold of the adaptive filter (the lower line) and the signal level threshold (the upper line). The highlighted areas are the positions where the Pan and Tompkins methods make inaccurate detection of the QRS complex. This inaccuracy can be noticed where the circles in Fig 15(A) and the intermittent lines in Fig 15(B) are congested. However, after applying the proposed algorithm, the detection accuracy is improved. This improvement is highlighted in Fig 15(C) and 15(D) where it is clear that the stuffed circles and lines have been removed. The reason for this improvement is that the proposed HFBE and RHFBE algorithms can accurately estimate the correct position of the R-peak during segmentation process after the raw ECG signal had been filtered using the preprocessing module described earlier.

In conclusion, statistically-based filtering such as the Hampel filter shows its advantage in filtering ECG signals contaminated by motion artefact type noises. This method can be used for filtering many signals that have repetitive structures, such as respiratory signals. The advantage of the proposed Hampel filter-based method is that it is real-time realizable because it is based on a statistical approach. The potential issue with the proposed method is that if the QRS complex is totally contaminated, then the motion artefact in that particular ECG segment will not be recovered. To remedy this issue, the occurrence of the ECG segment e.g. the R-peak should be predicted by analyzing the the signal structure of the subsequent cardiac cycles. This is a trivial task, due to the availability of knowledge about the maximum and minimum length of two successive R-peaks (R-R interval). The task also is easier in the sinus rhythm signals rather than arrhythmia signal structures.

It should also be mentioned that the proposed Hampel filter based estimation works based on the concept of adjusting the outliers samples in the recorded ECG signal through analyzing the spatial correlation of the adjacent P-QRS-T segments. Moreover, the presence of a P-QRS-T segment which is very different from its neighbor is an indication of motion artifact. This concept is promising to differenciate the motion artefact from the cardiac diseases. Many cardiac related diseases occur regularly in the subsequent P-QRS-T segments in the ECG. Thus, Hampel filter based will perform well as long as all the neighboring segments have similar patterns. However, can Hampel filter based estimation differentiate between the motion artifact and the abnormal ECG signal that contains irregular pattern such as in Premature ventricular contraction? Theoretically, Hampel filter is a conditional median filter, i.e., if the current P-QRS-T segments differ much from the adjacent segments, then Hampel filter will adjust only the outlier portion of the segment. For example, if the ECG sample exceeded a particular threshold, namely the *m*^*th*^ number of the median absolute deviation as illustrated in Eq (13), then Hampel filter will replace it by the median of the neighboring signals. This threshold can be manually adjusted by the experts to avoid removing disease pattern. A broad investigation should be carried on cardiac rhythm signals for different diseases. However, we have left this issue for the future work.

## Conclusion

Obtaining accurate ECG signals during a subject’s normal activities is recognized as one of the major challenges in automatic health diagnosis applications. Adaptive filtering using noise as a reference has received a great deal of attention from researchers in the last few years. Most of the reported works used an accelerometer or gyroscope as noise reference in adaptive filtering algorithms. However, this approach could not be effective, due to low correlation between the noise and the acceleration signal. Adaptive noise cancelling using a low correlated noise reference causes the addition of more noise to the filtered signal, which leads to more filtering challenges. In this paper, we have proposed two filtering methods. The first method is based on adaptive filtering algorithms by proposing a weighted adaptive noise cancelling method. The correlation between the ECG and noise reference is used to degrade the impact of wrong parameters. The second method is estimation of the ECG signal itself using a Hampel filter. This method assumes that the recorded point that is contaminated with a motion artefact is considered as an outlier with respect to the location of the same point in the successive ECG segments. Thus, two-stage filtering using these two methods sequentially is carried out to solve the issue of the motion artefact in the ECG signal. The results show promising reductions of motion artefact noises from mobile ECGs during the subject’s different activities.

## Supporting information

S1 Supporting Information Compressed/ZIP File Archive Dataset contains the recorded vital signs and the body motion data (ZIP). (RAR)

S2 Supporting Information Compressed/ZIP File Archive ECG Results contains more details about the results presented in the manuscript (ZIP). (RAR)
